# Correlated nanoimaging of structure and dynamics of cation-polaron coupling in hybrid perovskites

**DOI:** 10.1126/sciadv.ads3706

**Published:** 2025-02-26

**Authors:** Roland Wilcken, Branden L. Esses, Rachith S. Nithyananda Kumar, Lauren A. Hurley, Sean E. Shaheen, Markus B. Raschke

**Affiliations:** ^1^ Department of Physics and JILA, University of Colorado, Boulder, CO 80309, USA.; ^2^ Department of Electrical, Computer, and Energy Engineering, University of Colorado, Boulder, CO 80309, USA.; ^3^ Institute for Materials Research (IMO-IMOMEC), Hasselt University, Agoralaan 1, 3590 Diepenbeek, Belgium.; ^4^ IMOMEC Division, IMEC, Wetenschapspark 1, 3590 Diepenbeek, Belgium.; ^5^ Renewable and Sustainable Energy Institute, University of Colorado, Boulder, CO 80303, USA.

## Abstract

Hybrid organic-inorganic perovskites exhibit high photovoltaic performance and other novel photonic functions. While polaron formation is believed to facilitate efficient carrier transport, the elementary processes of the underlying electron-lattice coupling are yet poorly understood because of the multiscale chemical and structural heterogeneities. Here, we resolve in combined ground- and excited-state spatiospectral ultrafast nanoimaging how structural characteristics are related to both molecular cation and polaron dynamics. We use the observed nanoscale spatial variations of the formamidinium (FA) cation transient vibrational blue shifts as a local probe of the nonlocal polaron-cation coupling. From the correlation with nanomovies of the polaron dynamics, we then infer how a softer more polarizable lattice supports stable polarons and longer-lived residual carriers. This, together with a relative intragrain homogeneity in contrast to high intergrain heterogeneity, suggests pathways for improved synthesis and device engineering, and that perovskite photonics performance is still far from any fundamental limits.

## Teaser

Nanoscale heterogeneity in perovskites for photovoltaics influences the photoinduced coupled carrier and polaron-lattice dynamics.

## INTRODUCTION

Triple-cation perovskites (TCPs) are organic-inorganic hybrid materials (*1*) that rose to prominence due to a range of attractive photophysical and optoelectronic properties like high photovoltaic efficiency, overall stability, relative ease of fabrication, and wide bandgap tunability (*2*–*4*). Because of their flexibility in composition and structure, they hold promise for numerous other applications beyond photovoltaics, like lasing (*5*), exciton condensation (*6*), single-photon emitters (*7*), photodetectors (*8*), or photocatalysis (*9*).

Despite the many favorable attributes of TCPs, their limited photostability under operating conditions still challanges commercial solar cell applications. Device degradation appears to be mostly due to photo- and thermal-induced deleterious chemical reactions and structural phase separation (*10*–*12*), which nucleate at the nanoscale and then progress into the bulk (*13*). This necessitates a fundamental understanding of heterogeneity not only in nanoscale structure but also of the dynamic evolution of the photoinduced excited-state with the associated electron-lattice interaction and polaron formation stabilizing the photoinduced carriers (*14*).

Since the recognition that substantial progress in optimizing the performance of perovskites relies on understanding and control of both its intrinsic and extrinsic nanoscale heterogeneities in structure and composition (*15*), almost every conceivable high–spatial resolution microscopy technique has been applied for its investigation. This includes the full range of optical microscopies, Kelvin probe microscopy, conductive atomic force microscopy (AFM), different electron microscopies (scanning electron microscopy, transmission electron microscopy, four-dimensional scanning transmission electron microscopy), micro–x-ray fluorescence, and other (*16*–*20*). However, these techniques can only resolve slow kinetic or static ground-state properties, yet the photophysical responses from low-fluence photovoltaics to high field lasing are by their nature excited-state dynamic properties.

All spectroscopies that can measure excited-state lattice and electron dynamics and their coupling (*21*–*25*) are limited to large-scale and ensemble-averaged measurements with low spatial resolution. However, it is precisely the coupled electron and lattice dynamics on the nanoscale where the photophysical properties and their limitations are defined. While progress has been made toward dynamic imaging with high spatial resolution, such as in scattering microscopy (stroboSCAT) (*26*), scanning probe microscopy (*27*, *28*), or photoluminescence spectroscopy (*29*) for imaging photo-induced carrier transport on nanosecond to microsecond time scales (*30*), simultaneous picosecond to femtosecond time-resolved nanoimaging of perovskites has remained difficult.

Ultrafast pump-probe IR *s*-SNOM (infrared scattering scanning near-field optical microscopy), as established recently, combines nanoscale imaging with ultrafast spectroscopy in the mid-IR (MIR) (*31*–*35*) and terahertz spectral range (*36*). Further, nano-FTIR (Fourier transform IR) *s*-SNOM provides access to spatial heterogeneity in structure and chemical composition (*37*–*39*). In the application to perovskites, while a qualitative nanoscale spatial heterogeneity in carrier relaxation dynamics could be resolved (*40*), the link between structural heterogeneities and the photoinduced coupled electron-lattice dynamics could not be established.

**Fig. 1.**
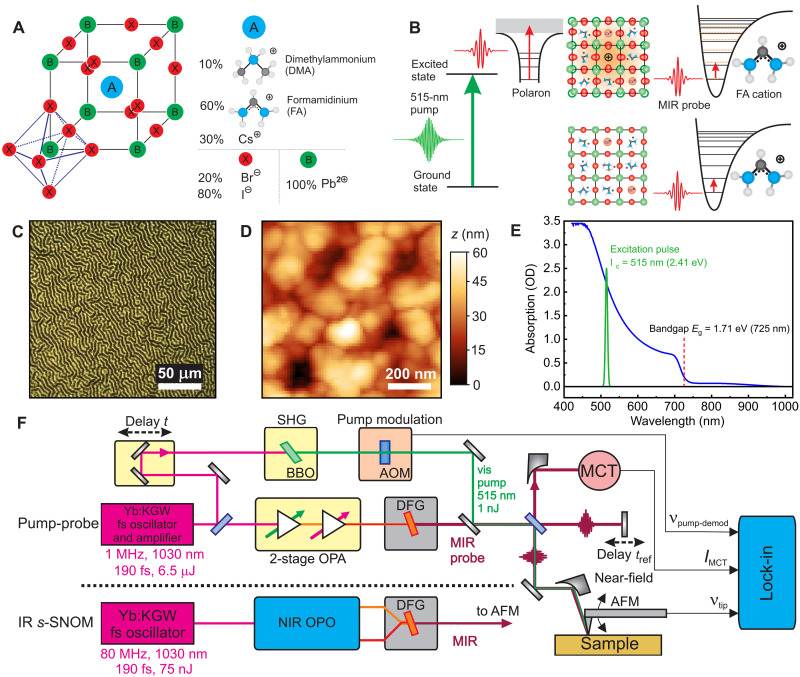
Ground-state structure and excited-state dynamics nanoimaging. (**A**) Structure and composition of the FADMACs TCP. (**B**) Above bandgap excitation (λ_pump_ = 515-nm pump and λ_bandgap_ = 725 nm) creates out-of-equilibrium carrier distribution. MIR pulses centered at $\bar{\nu }$ = 1715 cm^−1^ probe both the polaron absorption and the FA cation NCN vibration with and without pump excitation. (**C**) Optical microscope image with Turing pattern and (**D**) AFM image of the perovskite surface. (**E**) Absorption spectrum of the perovskite film (for bandgap determination, see the Supplementary Materials). (**F**) Schematic of the IR *s*-SNOM spatiospectral and heterodyne pump-probe spatiotemporal imaging (for details, see Materials and Methods).

In this work, we perform correlative spatio-spectral-temporal nanoimaging, resolving both the vibrational ground and excited-state responses and how they influence the coupled polaron dynamics as shown in Fig. 1. We chose a composition of (FA_0.6_DMA_0.1_Cs_0.3_)Pb(I_2.4_Br_0.6_) (FADMACs), with formamidinium (FA) and dimethylammonium (DMA) cations, which provides improved stability under ambient conditions and photostability compared to methylammonium-containing perovskites as reported recently (*41*). We observe grain-to-grain heterogeneity in the FA vibrational characteristics, which we attribute to variations in the local cation composition and associated changes in lattice structure (*42*), in contrast to a comparatively homogeneous intragrain response. The intergrain heterogeneity is amplified in the excited-state characterized by a transient FA vibrational blue shift that we interpret as a reaction field–induced vibrational Stark shift associated with polaron formation. The observed correlations between the Stark shift and the fraction of residual carriers at >1 ns, as well as polaron lifetimes and ground-state structure and composition, could aid in guiding materials synthesis and device fabrication to improve the photophysical properties of perovskites.

### Experiment

For the experiments, thin films of FADMACs perovskite were prepared by spin coating on borosilicate glass substrates as established (for details, see Materials and Methods) (*43*, *44*). The samples were precharacterized by optical absorption spectroscopy verifying the bandgap of 1.71 eV and AFM showing the typical surface texture composed of ~50- to 100-nm-sized grains (see Fig. 1, C to E).

We then perform static broadband nano-FTIR spatiospectral imaging of the FA cation vibration at ~1715 cm^−1^ to resolve nanoscale heterogeneity as previously established (*37*, *38*, *45*). We subsequently perform dynamic excited-state nano-FTIR spectroscopy at select sample locations following femtosecond visible pump (515 nm, 2.4 eV) excitation. Last, we perform and compare with full spatiotemporal pump-probe nanoimaging of the mid-IR polaron response (for further details see Materials and Methods) (*33*, *34*, *40*). The near-field spectra represent the complex valued dielectric response of the material expressed as near-filed amplitude *A*_NF_ and phase Φ_NF_ (corresponding to the absorption spectrum) according to ${\text{Re}}_{\text{NF}}\left(\bar{\nu }\right)+i{\text{Im}}_{\text{NF}}\left(\bar{\nu }\right)=A_{\text{NF}}\left(\bar{\nu }\right)\cdot e^{i\Phi_{\text{NF}}\left(\bar{\nu }\right)}$ (*38*).

## RESULTS

### Ground-state chemical and structural heterogeneity

**Fig. 2.**
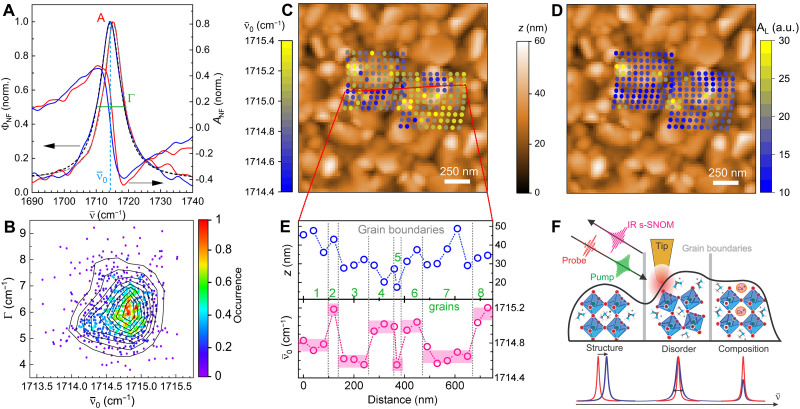
Ground-state chemical and structural heterogeneity. (**A**) Normalized ground-state IR *s*-SNOM spectral amplitude *A*_NF_ and phase Φ_NF_ of the antisymmetric stretch mode of the formamidinium (FA) cation on different grains, with Lorentzian fits to retrieve amplitude *A*_L_, center frequency ${\bar{\nu }}_{0}$, and FWHM linewidth Γ. (**B**) Scatter plot of Γ versus ${\bar{\nu }}_{0}$ of spectra from array scans on 10 different regions (~900 points in total) showing heterogeneity but no correlation. (**C** and **D**) 1.5 × 1.5 μm AFM topography overlaid results from spatiospectral array scans of center frequency ${\bar{\nu }}_{0}$ (C) and amplitude *A*_L_ (D) of the FA absorption. (**E**) Transect of ${\bar{\nu }}_{0}$ and AFM height, showing relative intragrain homogeneity and large intergrain heterogeneity. (**F**) Illustration of the influence of structure, disorder, and composition on nanoscale spectral behavior.

In the following section, we first describe the results of nano-IR spatiospectral imaging on the FADMACs perovskite films resolving the heterogeneity of the FA cation vibrational response in the electronic ground state. Figure 2A shows two representative FA point spectra, expressed as near-field spectral phase Φ_NF_ and amplitude *A*_NF_ fitted as single Lorentz oscillators. From the analysis of 900 spectra on different regions on the sample (Fig. 2B), we find that peak position and linewidth vary around ${\bar{\nu }}_{0}$ = (1714.7 ± 0.4) cm^−1^ and Γ = (6.2 ± 1.1) cm^−1^. No correlation in peak position ${\bar{\nu }}_{0}$ and linewidth Γ is observed and other parameters are only weakly correlated (see the Supplementary Materials; this contrasts previous findings and is further discussed below).

Figure 2 (C and D) shows AFM images with the typical FADMACs film texture with ~100-nm-size grains and ~50-nm-height variations, overlaid by two nano-FTIR array scans of ${\bar{\nu }}_{0}$ (C) and spectral phase amplitude *A*_L_ (D), where *A*_L_ of the near-field phase Φ_NF_ is a measure of the FA concentration. The amplitude *A*_L_ of the FA absorption exhibits spatial variation from 10° to 30° (see Fig. 2D), with the highest values all located on specific grains, while the majority of grains fall within ±5°. As highlighted by the transect in Fig. 2E, ${\bar{\nu }}_{0}$ shows a large degree of intergrain heterogeneity, yet comparably small intragrain spatial variation, even in the presence of topographic variations.

The underlying compositional variation is thus only weakly correlated with topography (see fig. S3C). Further, we see a weak positive correlation of amplitude *A*_L_ with peak position ${\bar{\nu }}_{0}$ and linewidth Γ (see fig. S3, B and D), which tend to shift to higher values with higher amplitude. We attribute the spectral blue shift to structural changes and a change in the reaction field affecting the FA cation caused by a local change in cation concentration between individual grains. In contrast, variations within a single grain are small compared to the entire ensemble (Fig. 2E). Within a single grain, ${\bar{\nu }}_{0}$ varies by ~0.1 cm^−1^ compared to ~0.4 cm^−1^ between neighboring grains.

We attribute this intergrain heterogeneity in ${\bar{\nu }}_{0}$ to a combination of local variations in both structure (defects and lattice deformation) and chemical composition as illustrated in Fig. 2F. The peak position ${\bar{\nu }}_{0}$ and the linewidth Γ relate to the reaction field modified by the local lattice structure as suggested previously (*37*), further discussed below. These heterogeneities have an even more pronounced effect on the excited-state dynamics as investigated in the next section.

### Excited-state dynamics of the FA vibration

**Fig. 3.**
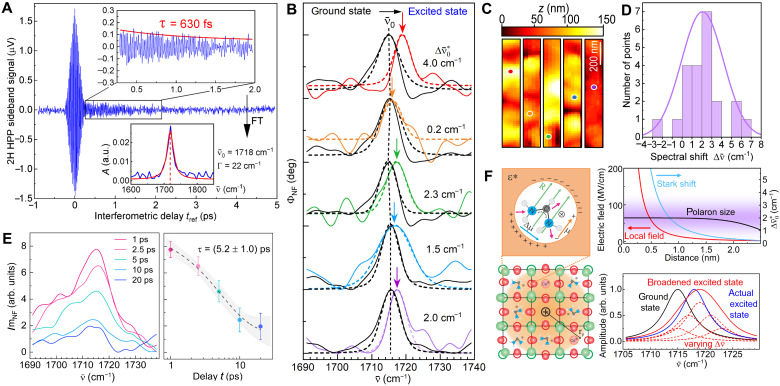
Excited-state transient vibrational nanospectroscopy. (**A**) Heterodyne pump-probe interferogram of the second-harmonic side band signal at *t* = 2 ps. (**B**) Pump-probe transient nano-FTIR phase Φ_NF_ spectra (solid, color) of the FA vibration for different grains of the perovskite film with Lorentzian fits (dashed), compared with corresponding ground-state spectra (black). (**C**) AFM topography with point spectra positions marked (scale bar, 200 nm). (**D**) Statistical distribution of the transient spectral shifts relative to ground state for these and other grains. (**E**) Pump-probe transient nano-FTIR Im_NF_ spectra measured at a single grain at *t* = 1, 2.5, 5, 10, and 20 ps (left), and signal decay with single exponential fit with time constant τ = (5.2 ± 1) ps (right). (**F**) FA cation with static dipole moment μ, within the polarizable continuum ε*, and resulting reaction field R (top left), induced by lattice deformation associated with large polaron formation within the polaron radius *r*_p_ (bottom left). Estimate (top right) of corresponding vibrational Stark shift (blue) based on unscreened potential (red) and inside the polaron (black), with illustration of the observed distribution of spectral shifts (purple). Calculated vibrational spectra (bottom right), with heterogeneous broadening estimated for the distance-dependent Coulomb field (red) of an unscreened carrier, and no broadening for a uniform field within a polaron (blue), compared to the ground state (black).

Next, we discuss the results of the ultrafast pump-probe nano-FTIR spectroscopy to resolve the dynamic spatiospectral heterogeneity of the FA vibration in response to the pump-induced electronic carrier excitation. Figure 3A shows the resulting excited-state vibrational free induction decay trace averaged over 10 point-spectra at a vis-pump IR-probe time delay of *t* = 2 ps, with corresponding Fourier transform (lower inset). Compared to the ground-state response, the excited-state FA spectra show on average a spectral blue shift $\Delta {\bar{\nu }}_{0}^{*}$ (see Fig. 3B) that varies spatially between −2 and +7 cm^−1^ between sample locations (Fig. 3C) with an uncertainty of ±0.5 cm^−1^. The statistical distribution (Fig. 3D) of the observed spectral blue shift averages at $\Delta {\bar{\nu }}_{0}^{*}=2.5\;{\text{cm}}^{-1}$, a value that is much larger than the spectral variation observed in the ground state of $\Delta {\bar{\nu }}_{0}=0.4\;{\text{cm}}^{-1}$. In addition, the observed excited-state dynamics are sensitive to heterogeneities in both the perovskite structure and composition, as discussed below.

The signal amplitude (Im_NF_) of the excited-state spectra (Fig. 3E, left) acquired on an individual grain decays with a time constant of (5.2 ± 1.0) ps (Fig. 3E, right). This time scale is similar to the fast component of the spectrally averaged polaron decay with time constant τ_fast_ = 6 to 12 ps (see the next section and table S1). Therefore, carrier relaxation seems to be physically linked to the decay of the FA cation vibration.

From these results, we conclude that the excited-state carriers change the polarizability of the lattice by large polaron formation, giving rise to a transient solvatochromic FA vibrational blue shift due to a reaction field–induced Stark effect (see Discussion). Conversely, the outlying negative value could result from lattice configurations responding with a slight decrease in polarizability.

### Excited-state dynamics of the polaron response

**Fig. 4.**
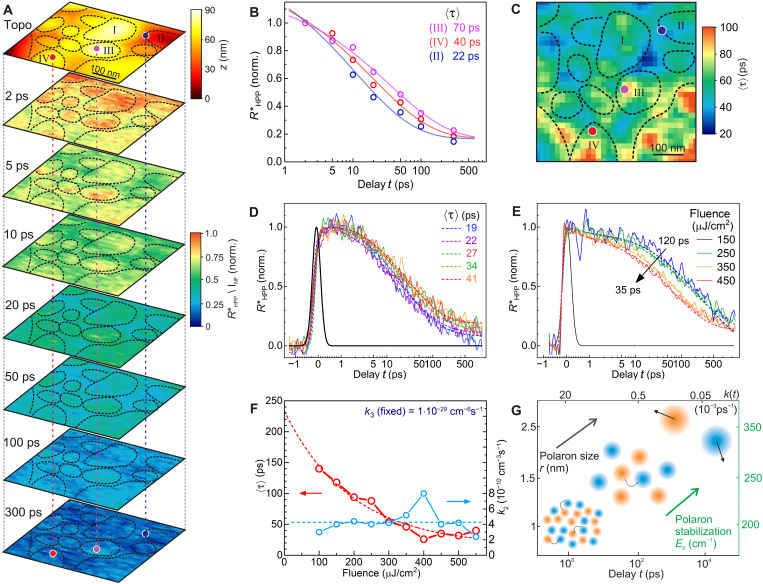
Excited-state carrier dynamics nanoimaging. (**A**) Two-phase heterodyne amplitude R^*^_HPP_ nanoimaging at seven pump-probe delay times (*t* = 2, 5, 10, 20, 50, 100, and 300 ps) compared to the topography (top). (**B**) Time traces at select locations from the nanoimage in color code (dots) fit with a stretched exponential function (solid lines). (**C**) Decay map of the imaging by fitting every pixel of the image with a stretched exponential resulting in an average time constant ⟨τ⟩ that demonstrates local variations in carrier recombination. (**D**) Two-phase heterodyne R^*^_HPP_ time traces (solid lines) scanning the pump-probe delay *t* at 5 locations on the surface (see Fig. 3B for related spectra) fitted by a stretched exponential. (**E**) Fluence-dependent time traces at a fixed location. (**F**) Time and rate constants from stretched exponential and rate model fits of the fluence-dependent decay curves. (**G**) Model for the polaron evolution over time with initial high density and small polaron radius with high recombination rates *k*(*t*) and increasing polaron size and stability over time [values for polaron size and binding energy from (*40*)].

We measure the polaron relaxation dynamics both at selected sample locations from above and in full spatiotemporal two-phase heterodyne nanoimaging. Figure 4A shows images of the spectrally integrated R^*^_HPP_/*I*_NF_ signal at variable delay times. The images are AFM drift corrected and normalized with respect to the second-harmonic ground-state image (i.e., pump pulse off) *I*_NF_ to account for small signal variations due to topography. With an excitation density of ~1 electron per 100 unit cells, corresponding to a volume of ca. 12.5 nm^3^, and the tip-localized near-field spatially averaging over ca. 10^4^ nm^3^, we probe about 10^3^ polarons per sample location or near-field image pixel.

Spatially heterogeneous decay dynamics varying on 10- to 100-nm spatial and 1- to 1000-ps temporal scales is observed. In this example, domains located in the upper (back) region of the image (e.g., grains I and II) exhibit a faster initial decay compared to domains in the lower (front) region (e.g., grains III and IV). This behavior is correlated with a larger (upper region) and smaller (lower region) signal level, respectively, at time zero.

From a simple qualitative inspection, a larger intergrain compared to intragrain variation in decay dynamics is clear, similar to the vibrational ground-state behavior. The range of the variation in decay dynamics is evident from the time traces at selected locations shown in Fig. 4B.

We then apply different models to analyze the decay dynamics quantitatively to describe the underlying polaron and carrier recombination processes. The high initial polaron density, the associated many-body interaction, and the increase in polaron size over time generally require a time-dependent rate equation model.

A stretched exponential model describes a time-dependent recombination rate without any prior assumptions of the underlying physical mechanism and provides an average time constant 〈τ〉 from the fitted stretched exponential time constant τ_SE_ and the stretching parameter β (*46*). The time evolution of the decay of the signal amplitude $R_{\text{HPP}}^{*}\left(t\right)$ can then be expressed as

$$R_{\text{HPP}}^{*}\left(t\right)=A_{0}\;e^{-{\left(\frac{t}{\tau_{\text{SE}}}\right)}^{\beta }}+y_{0}$$ (1)

with scaling factor *A*_0_ and offset *y*_0_. The average time constant 〈τ〉 is given by

$$\langle \tau \rangle =\tau_{\text{SE}}\Gamma \left(1+\frac{1}{\beta }\right)$$ (2)

with gamma function Γ. If the distribution underlying the carrier recombination probability is unknown, β can typically be fixed to 0.5 to adequately describe the time-dependent decay rate and to not overparameterize the fit (*47*). In this case, the average time constant is $\langle \tau \rangle =2\tau_{\text{SE}}$ (*46*).

Figure 4B shows fits (solid lines) to the data from selected locations using the stretched exponential model with 〈τ〉 = 22, 40, and 70 ps, respectively. Processing all image data in this fashion, Fig. 4C then shows the resulting spatiotemporal decay map of 〈τ〉 generated by stretched exponential fits to every pixel. As can be seen, 〈τ〉 ranges from ~20 to ~120 ps and reflects the spatial trends described above. Because of the limited number of time frames, the uncertainty for 〈τ〉 is estimated to be ~50%.

Further addressing variations in the decay dynamics, Fig. 4D shows R^*^_HPP_ time traces measured at the same locations as the pump-induced excited-state FA vibrational spectra in Fig. 3C. We add two additional exponentials to Eqs. 1 and 3 to account for both the initial polaron formation time τ_pol,_ and the long-lived residual carriers with an amplitude *R*_0_ with a lifetime on the nanosecond time scale responsible for the photovoltaic response. The final function is then numerically convoluted with the instrument response function (cross-correlation of pump and probe pulse) and fitted to the data. As can be seen in Fig. 4D, the signal peaks at ~1 ps with a rise time of τ_pol_ ~ 0.7 ps associated with hot carrier thermalization and polaron formation as established previously (*48*–*50*). Fitting with a stretched-exponential results in 〈τ〉 = 19 to 41 ps. The majority of the signal decays within 500 ps. Yet, a spatially variable residual signal remains even for *t* > 800 ps.

We further measured the pump-fluence dependence of the decay dynamics (Fig. 4E). For an increase of the fluence from 100 to 550 μJ/cm^2^ (pulse energy, 0.1 nJ to 0.55 nJ), a clear excitation-induced acceleration of the relaxation is observed with 〈τ〉 decreasing from 120 to 35 ps (dashed line). The acceleration seems to saturate with fluence at 〈τ〉 ~ 20 ps. An exponential extrapolation to zero fluence results in 〈τ〉_0_ ~ 240 ps. This value is still at least one order of magnitude faster than the carrier lifetimes under solar irradiance conditions. This discrepancy can be rationalized by a carrier stabilization mechanism like the formation of large polarons. At early delay times, the sheer number of carriers and their interactions drive the recombination and inhibit carrier stabilization, while decreasing carrier density allows the polaron radius to increase and sufficiently stabilize the carriers as indicated by the associated increasing polaron binding energy as illustrated in Fig. 4G. The increasing polaron radius and the polaron stabilization energy is estimated from time-resolved IR spectroscopy from previous publications based on the Emin’s theory (*40*, *51*), and the momentary recombination rate is calculated from the gradient of a rate model fit for a typical decay curve.

The polaron hypothesis is supported by the observation of a residual signal of ~10% of initial carriers that survive after >800 ps. However, the longer-lived subpopulation of carriers may not be identical to the long-lived carriers generated under solar irradiation conditions responsible for the photovoltaic response, and there is currently still a conceptual gap in the distinction between the solar and the high-density excitation regimes, which we discuss below.

A previously established alternative to the stretched exponential model describes the polaron recombination via a rate equation model for the time-dependent carrier density *n*(*t*) based on a bimolecular recombination rate *k*_2_, and higher-order rate constant *k*_3_ describing many-body processes like the Auger recombination (*34*, *52*, *53*). We neglect the first-order rate constant of single carriers because the higher-order terms dominate in the high-fluence regime we operate in (*40*), and the single-carrier recombination due to, e.g., defects, has been shown to be marginal in these types of perovskites (*52*, *53*). The corresponding rate equation is then given by

$$\frac{dn\left(t\right)}{dt}=-k_{2}n^{2}\left(t\right)-k_{3}n^{3}\left(t\right)$$ (3)

with $n\left(t\right)\propto R_{\text{HPP}}^{*}\left(t\right)$.

The assumption of a high carrier density is justified even though initially only about 1 electron/hole per 100 unit cells is excited, the polarons have a significant radius of ~1 nm initially (volume of 64 unit cells for lattice constant ~0.5 nm), and grow to ~2.5 nm (volume of 125 unit cells) over time (*40*). Because of their high mobility, a high recombination rate, and strong interactions between polarons is expected.

We now fit the data in Fig. 4 (D and E) with the rate equation model (Eq. 3) with an initial carrier density of *n*_0_ = 6 · 10^19^ for a pulse energy of 0.3 nJ (fluence, 300 μJ/cm^2^) assuming a unity photon-to-carrier conversion efficiency. We determine the average rate constants to be *k*_2_ = (6 ± 4) · 10^−10^ cm^−3^ s^−1^ and *k*_3_ = (1 ± 1) · 10^−29^ cm^−6^ s^−1^ for the selected locations of Fig. 4D by fitting each decay curve with the rate model and determining the mean and SD (see table S1 for fit values). We see an increase in the rate constants at different locations, consistent with a decrease in the stretched exponential decay time. For the fluence-dependent data, we keep *k*_3_ fixed and find values of *k*_2_ ~ 4 · 10^−10^ cm^−3^ s^−1^, largely independent of pump fluence (see Fig. 4F). Further, we observe that the recombination rate constants vary spatially, possibly due to material-dependent (composition, structure, crystallinity, or defect density) differences in initial carrier density due to locally different absorption cross sections or differences in recombination rates.

### Correlations between ground- and excited-state vibrational response and carrier dynamics

Last, we analyze correlations between ground and excited-state parameters (figs. S5 to S7) that can inform future material synthesis and device performance.

**Fig. 5.**
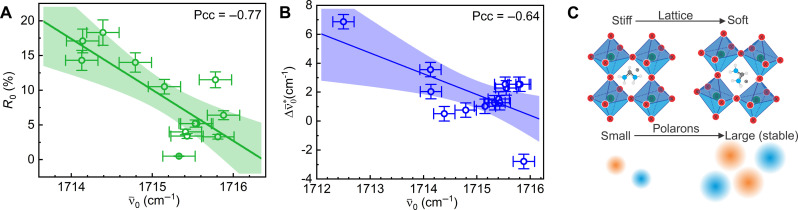
Correlations between ground and excited-state characteristics. (**A**) Fraction of remaining carriers 1 ns after excitation (*R*_0_), and (**B**) excited-state spectral shift $\Delta {\bar{\nu }}_{0}^{*}$ versus ground-state vibrational frequency ${\bar{\nu }}_{0}$. Trend line (linear fit, solid line) and 95% confidence interval (shaded area). Pearson correlation coefficient (Pcc) shows the degree of correlation (0: no correlation, 1 or −1: strong correlation). (**C**) Illustration for how a softer and more deformable lattice can foster more efficient polaron formation, in turn inducing a stronger reaction field–induced vibrational Stark shift.

Crucially, we observe correlations between ground-state peak position ${\bar{\nu }}_{0}$ of the FA vibration and (i) the residual carrier population *R*_0_ on the one hand (Fig. 5A) and (ii) the vibrational Stark shift $\Delta {\bar{\nu }}_{0}^{*}$ on the other (Fig. 5B). The ground-state vibrational red shift of the FA vibration can be viewed as a local reporter of a sparser or more disordered molecular lattice (*54*). The lattice is then expected to be more deformable, and polarons can stabilize more easily and faster, and thus recombination becomes less likely (Fig. 5A). This would equally facilitate a large polaron related transient vibrational blue shift $\Delta {\bar{\nu }}_{0}^{*}$ due to a stronger reaction field–induced Stark effect (Fig. 5B). The interrelation of both effects is illustrated in Fig. 5C.

We further note that the FA cation concentration (as represented by amplitude *A*_L_) is not correlated to any parameter (see fig. S6, I to K), and therefore, the relative concentration of DMA and cesium cations seem to be more related to beneficial material properties. Likewise, the spectral linewidth indicating cation-lattice coupling (higher coupling leading to faster dephasing and therefore homogeneous broadening) and disorder (inhomogeneous broadening) is also uncorrelated with excited-state properties (see fig. S6, E to H). Neither seems relevant for carrier stabilization and lifetime. This suggests that disorder itself does not diminish photocarrier stabilization, which would help explain the relative defect tolerance of these materials to their photovoltaic performance.

## DISCUSSION

In the following sections, we correlate the different ground- and excited-state observations and discuss possible underlying physical mechanisms.

### Ground-state vibrational nanospectroscopy

The FADMACs samples exhibit the nanoscale polycrystalline and granular texture typical for spin-coated thin films. The comparably narrow distribution (Fig. 2, B to E) in IR *s*-SNOM spectral signatures indicates that both composition (from signal amplitude *A*_L_) and structure (peak position ${\bar{\nu }}_{0}$ and linewidth Γ) are quite homogeneous within individual grains yet are clearly distinct grain to grain.

In contrast to previous findings of an anticorrelation between peak position and linewidth attributed to chemical heterogeneity-induced cation-lattice disorder on a similar perovskite (*34*), our results show that the FADMACs are more homogeneous in these aspects, which may be linked to its higher stability. This interpretation is in line with recent developments (*55*), suggesting a trend that perovskites that are more homogeneous on the nanoscale with larger grains of higher crystallinity and potentially less defects [e.g., FAPEA lead iodine perovskite (FA_0.95_PEA_0.05_PbI_3_) with phenylethlyammonium (PEA)]. seem to exhibit improved photostability. Therefore, even for identical composition, modifying the material synthesis to yield larger grains optimized for a structure (order, disorder, or crystallinity) (*56*) with beneficial ground- and excited-state properties could be a viable route for photophysical performance improvement.

### Transient vibrational nanospectroscopy

Key evidence for the importance of nanoscale heterogeneity of the microscopic processes underlying the macroscopic photophysical performance derives from the transient vibrational nanospectroscopy. For the pump fluence of 100 μJ/cm^−2^, the carrier density is *n*_0_ ~ 10^19^ cm^−3^ corresponding to 1% excitation density, i.e., ~1 polaron per 100 unit cells. However, the initial transient FA vibrational signal is as large as ~10% of that of the ground state, indicating a nonlocal response affecting an ensemble of FA cations within the region of the polaron radius *r*_p_ (see Fig. 3F).

The polaron as a self-trapped phonon-dressed state with an associated ionic lattice distortion extends over multiple unit cells (large polaron, Fröhlich type) as confirmed by diffuse x-ray scattering (*20*). The observed isotropic Stark shift $\Delta {\bar{\nu }}_{0}^{*}$ can be described by the Onsager model, where the static dipole moment of the FA cations induces a reaction field in the lattice analogous to solutes in a polarizable solvent (*57*, *58*). As established (*59*), the back action of the polaron-associated deformation of the polarization field itself gives rise to the “large polaron,” i.e., the polaron changes the polarizability of the lattice, which then can be considered a polarizable continuum ε*. This then leads to a stronger reaction field within the polaron radius, giving rise to the transient Stark shift observed (see Fig. 3).

It should be noted that both electron and hole polarons are present after photoexcitation. Even though their lattice coupling, mobility, and effective mass are different (*25*, *60*), our measurements of the polaron dynamics cannot distinguish between the different species. The different polaron types may manifest themselves in different degrees of solvatochromic blue shift, but we do not see evidence of two distinct populations like a line broadening or splitting.

On the basis of an effective Stark tuning rate of 10.3 (MV/cm)/cm^−1^ (*61*), the observed VSE shift of $\Delta {\bar{\nu }}_{0}^{*}=-2-7\;{\text{cm}}^{-1}$ would require a local field of 0 to 60 MV/cm with the maximum value consistent with the field of an unscreened electron located 0.5 nm (Fig. 3F) (i.e., equivalent to the size of a unit cell) from a FA cation (*61*) (see the Supplementary Materials for corresponding field calculation).

The lattice distortions associated with the polaron extend over several unit cells yet decrease only slowly with radial distance (*14*). Therefore, the FA cations are subject to a uniform reaction field across the polaron area. This would explain the observation of a well-defined transient vibrational frequency shift without an apparent change in linewidth. The Stark shift within this model is independent of the molecular orientation (*57*, *58*) but isotropic already because of the random orientation of the FA cations due to high rotational mobility (*62*), which itself has been suggested to contribute to carrier stabilization (*63*). The intra- and intergrain variation in the spectral shift can then be rationalized by local structural differences, which give rise to both variable reaction fields and polarons of different sizes. This contrasts with a large inhomogeneous broadening and a weak local response in the excited state as would be expected in case of a large unscreened electric field gradient (see Fig. 3F), which we do not observe.

Alternatively, direct dipole-dipole coupling between the molecular vibration and the polaron may give rise to a hybrid polaron-vibration state with an associated vibrational blue shift as suggested previously (*40*). However, this process is unlikely due to the high coupling rate of ~50 cm^−1^ for a 6 cm^−1^ shift when compared to typical coupling constants (~20 cm^−1^) and the linewidth of the vibrational mode (~6 cm^−1^). Although dipole-dipole coupling may manifest itself in an increase of the oscillator strength of the vibration in the excited state by borrowing strength from the lattice, evidence for such an increased oscillator strength has been observed in time-resolved IR spectroscopy (*24*, *25*, *64*) as a ~10 times larger excited-state signal compared to the ground-state bleach. This effect would still be too small to achieve the hybridization of polaron and molecular vibration in the bare material, yet it provides a perspective for associated IR polaron-vibration polariton formation and control with the help of high-*Q* IR cavities.

### Polaron dynamics

Last, we discuss the polaron dynamics and their spatial heterogeneity as it pertains to the carrier recombination that ultimately relates to device performance. Following the pump excitation, it has been proposed that the short-lived excitons [due to their low binding energy of <*kT* (300 K) = 25 meV] (*48*, *65*) decay into free electrons and holes to subsequently most form polarons within ~1 ps (*48*, *49*) (Fig. 4G). The spatial heterogeneity observed in the subsequent polaron decay dynamics shows a higher degree of intra- and intergrain heterogeneity than both static ground-state and transient vibrational excited-state nanospectroscopy. This may be expected because the excitation density-dependent recombination can be a superposition of spatial variations in both absorptions, i.e., initial carrier density, and recombination rates, both of which are influenced by heterogeneity in local composition and structure (see fig. S5).

While the details about the true nature of charge carriers in perovskite are complex and discussions are still ongoing, some general conclusions can be drawn from the systematic trends observed. Foremost, the fluence-dependent measurements show that the initial momentary recombination rate scales with initial carrier density over a wide range of fluences. Increasing the pump fluence beyond 500 μJ/cm^2^ does not increase the recombination rate further. This has previously been attributed to a limit on polaron diffusion (*66*) or a hot-phonon bottleneck (*67*). Further, we observe nearly constant values for the nominal recombination rate constants describing the bimolecular recombination (*k*_2_) and Auger recombination (*k*_3_) (see Fig. 4F). Therefore, their values depend only on material properties and not on initial carrier density.

Increasing the pump fluence reduces the fraction of residual carrier density that survives into the nanosecond regime (see fig. S8). However, with further increasing fluence the value of *R*_0_ stabilizes at 10 to 20%. Overall, for photovoltaic devices, a lower initial carrier density is preferable, which results in a higher relative number of stabilized carriers. For high excitation applications like perovskite-based lasers, the number of usable carriers after 1 ns remains above 10% with higher fluence even though the initial carrier recombination becomes faster.

Our results support the interpretation that the soft ionic lattice plays a crucial role by reacting dynamically to the excited charge carriers (*20*, *36*, *68*–*70*) and favor the polaron model. Our results agree with previous transient absorption measurements evaluated on the basis the polaron model (*40*, *51*), which suggest that as the polaron density decreases, the polarons grow in size, and simultaneously the polaron binding energy increases, which leads to a stabilization of the charge carriers (see Fig. 4G). However, it should be emphasized that the relationship between the >1 ns residual carrier population after a high-density laser excitation and the carrier population excited under solar irradiance conditions has not yet been established.

### Summary and perspective

In summary, we performed combined ground- and ultrafast excited-state IR nanoimaging of FADMACs as one of the more stable metal halide perovskites and promising candidates for tandem solar cells, photocatalysis, and optoelectronic applications. The multiparameter analysis provided systematic insight into the outstanding question of the influence of ground-state heterogeneity on excited-state coupled transient vibrational and polaron dynamics, resolving differences in intra- versus intergrain heterogeneity conventionally lost in spatially averaged spectroscopy.

The correlative analysis reveals that a softer lattice characterized by a red-shifted FA ground-state vibration leads to a larger number of long-lived carriers due to enhanced polaron stabilization in the transition from an initial picosecond nonlinear to a nanosecond linear recombination regime associated with polaron size evolution. A correspondingly large transient FA response with signals 10-times larger than expected compared to the excited state carrier density alone, together with a large blue shift of the FA vibration then signify the formation of larger and more stable polarons. We rationalize the uniform vibrational Stark shift by an Onsager-type reaction field model independent of the molecular orientation of the cation reporter vibration, thus sensing the polaron-induced uniform change of the lattice polarizability and the resulting vibrational reaction field of all cations within the polaron radius.

This work thus highlights the ability of the transient vibrational solvatochromism of the FA vibration to serve as a local probe of the nonlocal polaron formation, and a key potential metric in correlative analysis of the compounding factors linking microscopic heterogeneity with macroscopic photophysical device performance.

As a perspective, our work shows how correlated ground- and excited-state structural and dynamic nanoimaging could guide optimization of composition and thin film preparation. Larger grains of a composition or structure optimized for long-lived carriers are expected to improve photovoltaic device performance in the low-fluence regime to, e.g., lasing applications at high excitation fluences.

With that, we have demonstrated a pathway toward resolving the missing links between heterogeneity in composition, structure, and disorder to photovoltaic device performance and stability. In future work, more correlative nanospectroscopy and nanoimaging would be needed to include chemical nanoimaging, on a real device in operando to relate microscopic composition, structure, coupling, and dynamics to better understand the complex time evolution of polarons and their impact on the macroscopic device performance.

## MATERIALS AND METHODS

### FADMACs film preparation

To prepare (FA_0.6_DMA_0.1_Cs_0.3_)Pb(I_2.4_Br_0.6)_ perovskite films, precursor salts (PbI_2_ = 322.7 mg, PbBr_2_ = 110.1 mg, FA-iodine = 103.2 mg, CsI = 77.9 mg, and DMA-iodine = 17.3 mg) were dissolved in 1 ml of *N*,*N*′-dimethylformamide:dimethyl sulfoxide 3:1 (volume ratio) to obtain a 1 M solution. The solution was prepared in an inert atmosphere (glove box with dry N_2_) and stirred overnight (14 hours) at room temperature. Addition of DMA leads to an increased bandgap and a larger octahedral tilt. Although the exact mechanism is still undetermined, it results in better photostability due to reduced photoinduced halide phase separation, and the increased bandgap is beneficial for tandem solar cells (>1.7 eV) (*43*, *44*).

Borosilicate glass substrates (2 × 2 cm^2^) were cleaned by sonication in trichlorormethane, acetone, and methanol. The perovskite films were grown by spin coating onto the substrates under inert atmosphere. About 200 μl of the solution were pipetted onto the substrate, distributed with the pipette tip, and the spin coater was first run at 2000 rpm for 20 s, then 6000 rpm for 40 s with inert gas stream quenching for the last 10 s. Films were subsequently annealed for 20 min at 120°C on a hot plate. The resulting films were black and opaque around the middle of the substrate and increasingly more transparent and inhomogeneous toward the edges.

These substrates were cut into smaller pieces under an inert atmosphere. Films were selected from center segments and taped on magnetic AFM plates. Samples were kept under inert gas until transferred into the AFM purged with nitrogen for pump-probe nanoimaging or under dry air for IR *s*-SNOM.

### Film characterization

Perovskite films were precharacterized using absorption spectroscopy, optical microscopy, and AFM. Figure S1A shows an absorption spectrum with the 1.71-eV (725-nm) bandgap determined using the Tauc method (*71*) (fig. S1B). A film thickness of ~300 nm is expected for our preparation method (*44*). Figure S1C shows an image of the film-coated substrate before cutting. Figure S1D shows a microscope image of the surface with the micrometer- to millimeter-size domains and ~100-nm-height modulation that results from the Marangoni effect during spin coating. The AFM image in fig. S1E shows ~100-nm-size grains and ~50-nm-height variation as typical for freshly prepared films of this type (*15*) of perovskite, while increasing grain size and growing roughness would indicate degradation.

### Ultrafast nanoimaging and IR *s*-SNOM

For the combined ground-state structure and excited-state dynamics nanoimaging, experiments are performed on two different IR *s*-SNOM setups, one for nano-FTIR spatiospectral imaging of the ground-state response, and one for ultrafast vis-pump IR-probe nanoimaging in the photoinduced excited state.

### Ground-state spatiospectral nano-IR imaging

IR *s*-SNOM (Fig. 1C) with interferometric heterodyne detection of the tip scattered near-field is performed as established previously (*37*, *38*). Experiments were conducted on a modified prototype IR *s*-SNOM system (Anasys NanoIR1-s, Bruker Nano Surface, Goleta, CA) using an optical parametric oscillator (OPO) followed by difference frequency generation (DFG) system (Levante/HarmoniXX, APE GmbH, Berlin) for tunable (5 to 10 μm) MIR pulse generation, pumped by a Yb:KGW (1030 nm and 1.2 eV) femtosecond oscillator (6.5 W, 80 MHz, and ~190 fs; FLINT, Light Conversion, Vilnius), directed onto a Pt/Ir-coated tip with ~250 kHz tapping frequency (ARROW-NCPt, NanoAndMore USA Corp., Watsonville). The OPO/DFG system is tuned to 5.8 μm center wavelength (1715 cm^−1^), delivering IR pulses of 250 fs pulse duration to probe the FA antisymmetric stretch mode at ${\bar{\nu }}_{0}$ = 1715 cm^−1^ in nano-FTIR with spatial-spectral resolution.

We measured the tip-scattered signal in back-reflection in heterodyne detection with an excitation pulse replica [local oscillator (LO)] in an asymmetric Michelson interferometer and detect the signal with a HgCdTe (MCT) detector (KLD 0.1-J1, Kolmar Technologies Inc., Newburyport). The signal is demodulated via lock-in detection (HF2LI, Zurich Instruments Ltd., Zurich) on harmonics of the tip tapping frequency ν_tip_ = 250 kHz (*n*ν_tip_ with *n* = 1, 2, 3...) to isolate the near-field signal *I*_NF_ ∝ *E*_NF_(ν_tip_)E*_LO_ with a spectral resolution of 1 cm^−1^ after the Fourier transform of the recorded interferogram. Spectra are phase corrected by polynomial fits to the spectral phase and normalized by complex division with a reference spectrum measured on germanium. The phase spectra are fitted by a Lorentz function and a single-layer point-dipole model (*72*). Amplitude *A*_L_, center frequency ${\bar{\nu }}_{0}$, and linewidth Γ determined by Lorentz oscillator fits report local composition and lattice structure in the ground state.

### Excited-state pump-probe spatio-spectral-temporal nanoimaging

The excited-state dynamics is resolved by pump-probe IR *s*-SNOM with heterodyne detection (HPP IR *s*-SNOM) as described previously (*34*). Figure 1C shows the home-built IR *s*-SNOM setup, based on a modified AFM (Innova, Bruker Nano Surface, Goleta, CA), using the same Pt/Ir tips (see above). The femtosecond laser pulses are focused onto the tip using an off-axis parabolic mirror (numerical aperture = 0.45, *f* = 5 cm, and ϕ = 25 mm, custom). The tip-scattered near-field IR light is again interferometrically heterodyne detected by an MCT (KLD 0.1-J1, Kolmar Technologies Inc., Newburyport) and lock-in demodulated (HF2LI, Zurich Instruments Ltd., Zurich) as described above, with similar spectral processing with the exception that the spectral phase Φ_NF_(${\bar{\nu }}_{0}$) of the side band demodulated spectrum (excited state) is phase corrected with the second-harmonic signal phase to account for a phase shift that carries information, e.g., about the phase offset from the broad polaron/Drude spectrum of the excited-state carriers.

A solid-state Yb:KGW oscillator/regenerative amplifier (6 W, 1 MHz, ~190 fs, and 1030 nm; Pharos, Light Conversion, Vilnius) is used to provide femtosecond pules, where a ~10% fraction (500 mW) of the fundamental power is frequency doubled to 515 nm (2.4 eV) in a BBO crystal to generate visible pulses for above bandgap excitation of the perovskite films. The remaining fundamental power drives an optical parametric amplifier followed by DFG, providing a tunable mid-IR probe pulse (5 to 10 μm, ~180 fs, and full width at half maximum = 150 cm^−1^). The path length of the visible pump pulse is adjusted via a mechanical delay line (CLL42, SmarAct GmbH, Oldenburg), producing a pump-probe time delay (*t*) of up to 1 ns.

The amplitude of the pump pulse is sinusoidally modulated by an acousto-optic modulator with frequency Ω_M_ = 16 kHz. This allows sideband detection of the lock-in amplified IR *s*-SNOM signal and isolation of the pump-induced signal at frequencies of *n*ν_tip_ ± Ω_M_ corresponding to the pump-induced change in the near-field signal Δ*I*_NF_(*t*) as a function of the pump-probe delay *t*. Scanning the interferometric delay (*t*_ref_) results in the heterodyne pump-probe (HPP) signal I^*^_HPP_(*t*, *t*_ref_) with the complex spectral response function of the excited state to $A_{\text{NF}}^{*}\left(\bar{\nu }\right)\cdot e^{i\Phi_{\text{NF}}^{*}\left(\bar{\nu }\right)}$ after Fourier transform.

Pump-pulse energies were varied from 0.1 to 0.5 nJ (power, 100 to 500 μW; and fluence, 100 to 500 μJ/cm^2^). A noticeable acceleration of the sample degradation with pump power greater than 500 μW was observed. Most experiments were performed at ~0.3 nJ as a compromise between signal level and measurement time before sample degradation (2 to 5 hours). Indicators of sample degradation include an increase in nanoscopic roughness of the sample surface, increase of grain size and decreased signal due to formation of lead halides and cation migration. The entire IR *s*-SNOM setup is fully enclosed and purged with dry nitrogen.

The probe pulse was centered at ${\bar{\nu }}_{\text{probe}}$ ~ 1715 cm^−1^ to cover both the FA spectrum and the excited carrier decay from the broad polaron response. Scanning the interferometric delay *t*_ref_ at a fixed pump-probe delay *t* provides both the ground-state spectrum from *n*ν_tip_ and the excited-state spectrum from the sideband modulated signal *n*ν_tip_ ± Ω_M_. Therefore, we obtain the FA vibrational response in both equilibrium and out-of-equilibrium states along with its corresponding relaxation dynamics.

Fixing the interferometer delay *t*_ref_ and scanning the pump-probe delay *t* allows us to extract the temporal dynamics of the excited state, discriminated from the self-homodyne background by measuring the signal amplitude *R*_HPP_(*t*) decay using two-phase heterodyne detection. The time delay *t*_ref_ is fixed at a delay corresponding to 0 and π phase of the HPP interferogram at the center burst while *t* is scanned twice, once at each fixed delay *t*_ref_. The difference of these signals then corresponds to the excited-state decay R*_HPP_(t) = I*_HPP_(*t*, 0) − I*_HPP_(*t*, π). Both decay curves are recorded at a fixed sample location, or at selected time delays, *t* images are recorded by sample scanning to R*_HPP_(*t*, *x*, *y*) = I*_HPP_(*t*, 0, *x*, *y*) − I*_HPP_(*t*, π, *x*, *y*).

Probing at ~1715 cm^−1^ and recording the second-harmonic near-field signal and its pump-induced change as a side band demodulated signal provides four datasets: the ground-state spectrum $A_{g}\left(\bar{\nu }\right)e^{i\phi_{g}\left(\bar{\nu }\right)}$ and the excited-state spectrum $A_{e}\left(\bar{\nu }\right)e^{i\phi_{e}\left(\bar{\nu }\right)}$ of the FA cation vibration (simultaneously, by scanning the reference arm delay *t*_ref_ at a fixed pump delay *t*), the pump-probe transient of the broadband polaron/Drude response by scanning the pump-probe time delay *t*, and a spatial array of these properties with nanoscale spatial resolution by sample scanning.

Fundamentally, measuring all these parameters simultaneously would require sequentially obtaining an interferogram (including several averages, ~30 s per scan, and ~6 cm^−1^ spectral resolution) at every time delay (at least 50 points logarithmically spaced point for meaningful time behavior) and at every image pixel in an AFM image (at least 50 points for a meaningful image). This would accumulate to a prohibitively long 21 hours.

Instead, while such full spatio-spectral-temporal information is in principle desirable, iteratively informed subsampling within this large spatio-temporal-spectral phase space is generally sufficient to extract the relevant physical information from the material system. Therefore, in an iterative informed process, we measure ground- and excited-state nano-IR spectra at specifically selected sample locations, two-phase heterodyne pump-probe transients at the same locations, and spectrally integrated nanoimaging at selected time points.

## References

[1] M. Saliba, T. Matsui, J.-Y. Seo, K. Domanski, J.-P. Correa-Baena, M. K. Nazeeruddin, S. M. Zakeeruddin, W. Tress, A. Abate, A. Hagfeldt, M. Grätzel, Cesium-containing triple cation perovskite solar cells: Improved stability, reproducibility and high efficiency. Energy Environ. Sci. 9, 1989–1997 (2016). 27478500 10.1039/c5ee03874j PMC4936376

[2] A. Kojima, K. Teshima, Y. Shirai, T. Miyasaka, Organometal halide perovskites as visible-light sensitizers for photovoltaic cells. J. Am. Chem. Soc. 131, 6050–6051 (2009). 19366264 10.1021/ja809598r

[3] H.-S. Kim, C.-R. Lee, J.-H. Im, K.-B. Lee, T. Moehl, A. Marchioro, S.-J. Moon, R. Humphry-Baker, J.-H. Yum, J. E. Moser, M. Grätzel, N.-G. Park, Lead iodide perovskite sensitized all-solid-state submicron thin film mesoscopic solar cell with efficiency exceeding 9%. Sci. Rep. 2, 591 (2012). 22912919 10.1038/srep00591 PMC3423636

[4] A. K. Jena, A. Kulkarni, T. Miyasaka, Halide perovskite photovoltaics: Background, status, and future prospects. Chem. Rev. 119, 3036–3103 (2019). 30821144 10.1021/acs.chemrev.8b00539

[5] L. Lei, Q. Dong, K. Gundogdu, F. So, Metal halide perovskites for laser applications. Adv. Funct. Mater. 31, 2010144 (2021).

[6] R. Su, S. Ghosh, J. Wang, S. Liu, C. Diederichs, T. C. H. Liew, Q. Xiong, Observation of exciton polariton condensation in a perovskite lattice at room temperature. Nat. Phys. 16, 301–306 (2020).

[7] M. Esmann, S. C. Wein, C. Antón-Solanas, Solid-state single-photon sources: Recent advances for novel quantum materials. Adv. Funct. Mater. 34, 2315936 (2024).

[8] H. Wang, D. H. Kim, Perovskite-based photodetectors: Materials and devices. Chem. Soc. Rev. 46, 5204–5236 (2017). 28795697 10.1039/c6cs00896h

[9] H. Huang, B. Pradhan, J. Hofkens, M. B. J. Roeffaers, J. A. Steele, Solar-driven metal halide perovskite photocatalysis: Design, stability, and performance. ACS Energy Lett. 5, 1107–1123 (2020).

[10] D. W. deQuilettes, W. Zhang, V. M. Burlakov, D. J. Graham, T. Leijtens, A. Osherov, V. Bulović, H. J. Snaith, D. S. Ginger, S. D. Stranks, Photo-induced halide redistribution in organic–inorganic perovskite films. Nat. Commun. 7, 11683 (2016). 27216703 10.1038/ncomms11683 PMC4890321

[11] F. Jiang, J. Pothoof, F. Muckel, R. Giridharagopal, J. Wang, D. S. Ginger, Scanning kelvin probe microscopy reveals that ion motion varies with dimensionality in 2d halide perovskites. ACS Energy Lett. 6, 100–108 (2021).

[12] M. Lai, A. Obliger, D. Lu, C. S. Kley, C. G. Bischak, Q. Kong, T. Lei, L. Dou, N. S. Ginsberg, D. T. Limmer, P. Yang, Intrinsic anion diffusivity in lead halide perovskites is facilitated by a soft lattice. Proc. Nat. Acad. Sci. U.S.A. 115, 11929–11934 (2018). 10.1073/pnas.1812718115 PMC6255190 30397127

[13] C. G. Bischak, C. L. Hetherington, H. Wu, S. Aloni, D. F. Ogletree, D. T. Limmer, N. S. Ginsberg, Origin of reversible photoinduced phase separation in hybrid perovskites. Nano Lett. 17, 1028–1033 (2017). 28134530 10.1021/acs.nanolett.6b04453

[14] C. Franchini, M. Reticcioli, M. Setvin, U. Diebold, Polarons in materials. Nat. Rev. Mater. 6, 560–586 (2021).

[15] E. M. Tennyson, T. A. S. Doherty, S. D. Stranks, Heterogeneity at multiple length scales in halide perovskite semiconductors. Nat. Rev. Mater. 4, 573–587 (2019).

[16] B. Guzelturk, T. Winkler, T. W. J. Van de Goor, M. D. Smith, S. A. Bourelle, S. Feldmann, M. Trigo, S. W. Teitelbaum, H.-G. Steinrück, G. A. de la Pena, R. Alonso-Mori, D. Zhu, T. Sato, H. I. Karunadasa, M. F. Toney, F. Deschler, A. M. Lindenberg, Visualization of dynamic polaronic strain fields in hybrid lead halide perovskites. Nat. Mater. 20, 618–623 (2021). 33398119 10.1038/s41563-020-00865-5

[17] S. T. Birkhold, J. T. Precht, R. Giridharagopal, G. E. Eperon, L. Schmidt-Mende, D. S. Ginger, Direct observation and quantitative analysis of mobile Frenkel defects in metal halide perovskites using scanning kelvin probe microscopy. J. Phys. Chem. C 122, 12633–12639 (2018).

[18] H. Si, S. Zhang, S. Ma, Z. Xiong, A. Kausar, Q. Liao, Z. Zhang, A. Sattar, Z. Kang, Y. Zhang, Emerging conductive atomic force microscopy for metal halide perovskite materials and solar cells. Adv. Energy Mater. 10, 1903922 (2020).

[19] Y. Zhou, H. Sternlicht, N. P. Padture, Transmission electron microscopy of halide perovskite materials and devices. Joule 3, 641–661 (2019).

[20] S. Wieghold, E. M. Cope, G. Moller, N. Shirato, B. Guzelturk, V. Rose, L. Nienhaus, Stressing halide perovskites with light and electric fields. ACS Energy Lett. 7, 2211–2218 (2022).

[21] M. Park, A. J. Neukirch, S. E. Reyes-Lillo, M. Lai, S. R. Ellis, D. Dietze, J. B. Neaton, P. Yang, S. Tretiak, R. A. Mathies, Excited-state vibrational dynamics toward the polaron in methylammonium lead iodide perovskite. Nat. Commun. 9, 2525 (2018). 29955070 10.1038/s41467-018-04946-7 PMC6023914

[22] K. T. Munson, E. R. Kennehan, G. S. Doucette, J. B. Asbury, Dynamic disorder dominates delocalization, transport, and recombination in halide perovskites. Chem 4, 2826–2843 (2018).

[23] K. T. Munson, E. R. Kennehan, J. B. Asbury, Structural origins of the electronic properties of materials via time-resolved infrared spectroscopy. J. Mater. Chem. C Mater. 7, 5889–5909 (2019).

[24] V. C. A. Taylor, D. Tiwari, M. Duchi, P. M. Donaldson, I. P. Clark, D. J. Fermin, T. A. A. Oliver, Investigating the role of the organic cation in formamidinium lead iodide perovskite using ultrafast spectroscopy. J. Phys. Chem. Lett. 9, 895–901 (2018). 29389137 10.1021/acs.jpclett.7b03296

[25] D. Sandner, K. Sun, A. Stadlbauer, M. W. Heindl, Q. Y. Tan, M. Nuber, C. Soci, R. Kienberger, P. Müller-Buschbaum, F. Deschler, H. Iglev, Hole localization in bulk and 2D lead-halide perovskites studied by time-resolved infrared spectroscopy. J. Am. Chem. Soc. 146, 19852–19862 (2024). 38982763 10.1021/jacs.4c02958 PMC11273617

[26] H. L. Weaver, C. M. Went, J. Wong, D. Jasrasaria, E. Rabani, H. A. Atwater, N. S. Ginsberg, Detecting, distinguishing, and spatiotemporally tracking photogenerated charge and heat at the nanoscale. ACS Nano 17, 19011–19021 (2023). 37721430 10.1021/acsnano.3c04607 PMC10569093

[27] R. Giridharagopal, G. E. Rayermann, G. Shao, D. T. Moore, O. G. Reid, A. F. Tillack, D. J. Masiello, D. S. Ginger, Submicrosecond time resolution atomic force microscopy for probing nanoscale dynamics. Nano Lett. 12, 893–898 (2012). 22248070 10.1021/nl203956q

[28] D. U. Karatay, J. S. Harrison, M. S. Glaz, R. Giridharagopal, D. S. Ginger, Fast time-resolved electrostatic force microscopy: Achieving sub-cycle time resolution. Rev. Sci. Instrum. 87, 053702 (2016). 27250430 10.1063/1.4948396

[29] S. Jariwala, R. E. Kumar, G. E. Eperon, Y. Shi, D. P. Fenning, D. S. Ginger, Dimethylammonium addition to halide perovskite precursor increases vertical and lateral heterogeneity. ACS Energy Lett. 7, 204–210 (2022).

[30] S. Berweger, F. Zhang, B. W. Larson, A. J. Ferguson, A. F. Palmstrom, O. G. Reid, T. M. Wallis, K. Zhu, J. J. Berry, P. Kabos, S. U. Nanayakkara, Nanoscale photoexcited carrier dynamics in perovskites. J. Phys. Chem. Lett. 13, 2388–2395 (2022). 35257587 10.1021/acs.jpclett.2c00233

[31] M. Wagner, Z. Fei, A. S. McLeod, A. S. Rodin, W. Bao, E. G. Iwinski, Z. Zhao, M. Goldflam, M. Liu, G. Dominguez, M. Thiemens, M. M. Fogler, A. H. Castro Neto, C. N. Lau, S. Amarie, F. Keilmann, D. N. Basov, Ultrafast and nanoscale plasmonic phenomena in exfoliated graphene revealed by infrared pump–probe nanoscopy. Nano Lett. 14, 894–900 (2014). 24479682 10.1021/nl4042577

[32] M. Plankl, P. E. Faria Junior, F. Mooshammer, T. Siday, M. Zizlsperger, F. Sandner, F. Schiegl, S. Maier, M. A. Huber, M. Gmitra, J. Fabian, J. L. Boland, T. L. Cocker, R. Huber, Subcycle contact-free nanoscopy of ultrafast interlayer transport in atomically thin heterostructures. Nat. Photonics 15, 594–600 (2021).

[33] S. A. Dönges, O. Khatib, B. T. O’Callahan, J. M. Atkin, J. H. Park, D. Cobden, M. B. Raschke, Ultrafast nanoimaging of the photoinduced phase transition dynamics in VO_2_. Nano Lett. 16, 3029–3035 (2016). 27096877 10.1021/acs.nanolett.5b05313

[34] J. Nishida, S. C. Johnson, P. T. S. Chang, D. M. Wharton, S. A. Dönges, O. Khatib, M. B. Raschke, Ultrafast infrared nano-imaging of far-from-equilibrium carrier and vibrational dynamics. Nat. Commun. 13, 1083 (2022). 35228517 10.1038/s41467-022-28224-9 PMC8885862

[35] G. X. Ni, L. Wang, M. D. Goldflam, M. Wagner, Z. Fei, A. S. McLeod, M. K. Liu, F. Keilmann, B. Özyilmaz, A. H. Castro Neto, J. Hone, M. M. Fogler, D. N. Basov, Ultrafast optical switching of infrared plasmon polaritons in high-mobility graphene. Nat. Photonics 10, 244–247 (2016).

[36] M. Zizlsperger, S. Nerreter, Q. Yuan, K. B. Lohmann, F. Sandner, F. Schiegl, C. Meineke, Y. A. Gerasimenko, L. M. Herz, T. Siday, M. A. Huber, M. B. Johnston, R. Huber, In situ nanoscopy of single-grain nanomorphology and ultrafast carrier dynamics in metal halide perovskites. Nat. Photonics 18, 975–981 (2024).

[37] J. Nishida, A. H. Alfaifi, T. P. Gray, S. E. Shaheen, M. B. Raschke, Heterogeneous cation–lattice interaction and dynamics in triple-cation perovskites revealed by infrared vibrational nanoscopy. ACS Energy Lett. 5, 1636–1643 (2020).

[38] E. A. Muller, B. Pollard, M. B. Raschke, Infrared chemical nano-imaging: Accessing structure, coupling, and dynamics on molecular length scales. J. Phys. Chem. Lett. 6, 1275–1284 (2015). 26262987 10.1021/acs.jpclett.5b00108

[39] F. Huth, A. Govyadinov, S. Amarie, W. Nuansing, F. Keilmann, R. Hillenbrand, Nano-FTIR absorption spectroscopy of molecular fingerprints at 20 nm spatial resolution. Nano Lett. 12, 3973–3978 (2012). 22703339 10.1021/nl301159v

[40] J. Nishida, P. T. S. Chang, J. Y. Ye, P. Sharma, D. M. Wharton, S. C. Johnson, S. E. Shaheen, M. B. Raschke, Nanoscale heterogeneity of ultrafast many-body carrier dynamics in triple cation perovskites. Nat. Commun. 13, 6582 (2022). 36323659 10.1038/s41467-022-33935-0 PMC9630529

[41] G. E. Eperon, K. H. Stone, L. E. Mundt, T. H. Schloemer, S. N. Habisreutinger, S. P. Dunfield, L. T. Schelhas, J. J. Berry, D. T. Moore, The role of dimethylammonium in bandgap modulation for stable halide perovskites. ACS Energy Lett. 5, 1856–1864 (2020).

[42] D. Ghosh, A. R. Smith, A. B. Walker, M. S. Islam, Mixed A-cation perovskites for solar cells: Atomic-scale insights into structural distortion, hydrogen bonding, and electronic properties. Chem. Mater. 30, 5194–5204 (2018).

[43] G. E. Eperon, M. T. Hörantner, H. J. Snaith, Metal halide perovskite tandem and multiple-junction photovoltaics. Nat. Rev. Chem. 1, 0095 (2017).

[44] A. F. Palmstrom, G. E. Eperon, T. Leijtens, R. Prasanna, S. N. Habisreutinger, W. Nemeth, E. A. Gaulding, S. P. Dunfield, M. Reese, S. Nanayakkara, T. Moot, J. Werner, J. Liu, B. To, S. T. Christensen, M. D. McGehee, M. F. A. M. van Hest, J. M. Luther, J. J. Berry, D. T. Moore, Enabling flexible all-perovskite tandem solar cells. Joule 3, 2193–2204 (2019).

[45] B. Pollard, F. C. B. Maia, M. B. Raschke, R. O. Freitas, Infrared vibrational nanospectroscopy by self-referenced interferometry. Nano Lett. 16, 55–61 (2016). 26654680 10.1021/acs.nanolett.5b02730

[46] M. N. Berberan-Santos, E. N. Bodunov, B. Valeur, Mathematical functions for the analysis of luminescence decays with underlying distributions 1. Kohlrausch decay function (stretched exponential). Chem. Phys. 315, 171–182 (2005).

[47] C. F. Sailer, S. Thallmair, B. P. Fingerhut, C. Nolte, J. Ammer, H. Mayr, I. Pugliesi, R. de Vivie-Riedle, E. Riedle, A comprehensive microscopic picture of the benzhydryl radical and cation photogeneration and interconversion through electron transfer. ChemPhysChem 14, 1423–1437 (2013). 23554328 10.1002/cphc.201201057

[48] K. Miyata, T. L. Atallah, X.-Y. Zhu, Lead halide perovskites: Crystal-liquid duality, phonon glass electron crystals, and large polaron formation. Sci. Adv. 3, e1701469 (2017). 29043296 10.1126/sciadv.1701469 PMC5640380

[49] K. Miyata, D. Meggiolaro, M. T. Trinh, P. P. Joshi, E. Mosconi, S. C. Jones, F. De Angelis, X.-Y. Zhu, Large polarons in lead halide perovskites. Sci. Adv. 3, e1701217 (2017). 28819647 10.1126/sciadv.1701217 PMC5553817

[50] J. M. Frost, L. D. Whalley, A. Walsh, Slow cooling of hot polarons in halide perovskite solar cells. ACS Energy Lett. 2, 2647–2652 (2017). 29250603 10.1021/acsenergylett.7b00862 PMC5727468

[51] D. Emin, Optical properties of large and small polarons and bipolarons. Phys. Rev. B 48, 13691–13702 (1993). 10.1103/physrevb.48.13691 10007771

[52] M. T. Trinh, X. Wu, D. Niesner, X. Y. Zhu, Many-body interactions in photo-excited lead iodide perovskite. J. Mater. Chem. A Mater. 3, 9285–9290 (2015).

[53] C. Wehrenfennig, G. E. Eperon, M. B. Johnston, H. J. Snaith, L. M. Herz, High charge carrier mobilities and lifetimes in organolead trihalide perovskites. Adv. Mater. 26, 1584–1589 (2014). 24757716 10.1002/adma.201305172 PMC4722848

[54] T. P. Gray, J. Nishida, S. C. Johnson, M. B. Raschke, 2D vibrational exciton nanoimaging of domain formation in self-assembled monolayers. Nano Lett. 21, 5754–5759 (2021). 34156252 10.1021/acs.nanolett.1c01515

[55] K. Wang, J. Huo, L. Cao, P. Yang, P. Müller-Buschbaum, Y. Tong, H. Wang, Fully methylammonium-free stable formamidinium lead iodide perovskite solar cells processed under humid air conditions. ACS Appl. Mater. Interfaces 15, 13353–13362 (2023). 36853957 10.1021/acsami.2c23134

[56] Z. Chu, M. Yang, P. Schulz, D. Wu, X. Ma, E. Seifert, L. Sun, X. Li, K. Zhu, K. Lai, Impact of grain boundaries on efficiency and stability of organic-inorganic trihalide perovskites. Nat. Commun. 8, 2230 (2017). 29263379 10.1038/s41467-017-02331-4 PMC5738431

[57] L. Onsager, Electric moments of molecules in liquids. J. Am. Chem. Soc. 58, 1486–1493 (1936).

[58] B. Pollard, E. A. Muller, K. Hinrichs, M. B. Raschke, Vibrational nano-spectroscopic imaging correlating structure with intermolecular coupling and dynamics. Nat. Commun. 5, 3587 (2014). 24721995 10.1038/ncomms4587 PMC4071972

[59] J. T. Devreese, “SEMICONDUCTOR PHYSICS | Polarons”, in *Encyclopedia of Modern Optics*, R. D. Guenther, Ed. (Elsevier, 2005; https://linkinghub.elsevier.com/retrieve/pii/B0123693950006291), pp. 1–8.

[60] Y. Zhai, K. Wang, F. Zhang, C. Xiao, A. H. Rose, K. Zhu, M. C. Beard, Individual electron and hole mobilities in lead-halide perovskites revealed by noncontact methods. ACS Energy Lett. 5, 47–55 (2020).

[61] R. Rakowski, W. Fisher, J. Calbo, M. Z. Mokhtar, X. Liang, D. Ding, J. M. Frost, S. A. Haque, A. Walsh, P. R. F. Barnes, J. Nelson, J. J. van Thor, High power irradiance dependence of charge species dynamics in hybrid perovskites and kinetic evidence for transient vibrational stark effect in formamidinium. Nanomaterials 12, 1616 (2022). 35630839 10.3390/nano12101616 PMC9146680

[62] A. Mishra, M. A. Hope, M. Grätzel, L. Emsley, A complete picture of cation dynamics in hybrid perovskite materials from solid-state NMR spectroscopy. J. Am. Chem. Soc. 145, 978–990 (2023). 36580303 10.1021/jacs.2c10149 PMC9853870

[63] A. J. Neukirch, W. Nie, J.-C. Blancon, K. Appavoo, H. Tsai, M. Y. Sfeir, C. Katan, L. Pedesseau, J. Even, J. J. Crochet, G. Gupta, A. D. Mohite, S. Tretiak, Polaron stabilization by cooperative lattice distortion and cation rotations in hybrid perovskite materials. Nano Lett. 16, 3809–3816 (2016). 27224519 10.1021/acs.nanolett.6b01218

[64] K. T. Munson, G. S. Doucette, E. R. Kennehan, J. R. Swartzfager, J. B. Asbury, Vibrational probe of the structural origins of slow recombination in halide perovskites. J. Phys. Chem. C 123, 7061–7073 (2019).

[65] M. Baranowski, P. Plochocka, Excitons in metal-halide perovskites. Adv. Energy Mater. 10, 1903659 (2020).

[66] D. W. deQuilettes, K. Frohna, D. Emin, T. Kirchartz, V. Bulovic, D. S. Ginger, S. D. Stranks, Charge-carrier recombination in halide perovskites. Chem. Rev. 119, 11007–11019 (2019). 31496228 10.1021/acs.chemrev.9b00169

[67] Y. Yang, D. P. Ostrowski, R. M. France, K. Zhu, J. van de Lagemaat, J. M. Luther, M. C. Beard, Observation of a hot-phonon bottleneck in lead-iodide perovskites. Nat. Photonics 10, 53–59 (2016).

[68] T. J. S. Evans, K. Miyata, P. P. Joshi, S. Maehrlein, F. Liu, X.-Y. Zhu, Competition between hot-electron cooling and large polaron screening in CsPbBr_3_ perovskite single crystals. J. Phys. Chem. C 122, 13724–13730 (2018).

[69] M. Frenzel, M. Cherasse, J. M. Urban, F. Wang, B. Xiang, L. Nest, L. Huber, L. Perfetti, M. Wolf, T. Kampfrath, X.-Y. Zhu, S. F. Maehrlein, Nonlinear terahertz control of the lead halide perovskite lattice. Sci. Adv. 9, eadg3856 (2023). 37224256 10.1126/sciadv.adg3856 PMC10208573

[70] X.-Y. Zhu, V. Podzorov, Charge carriers in hybrid organic–inorganic lead halide perovskites might be protected as large polarons. J. Phys. Chem. Lett. 6, 4758–4761 (2015). 26575427 10.1021/acs.jpclett.5b02462

[71] P. Makuła, M. Pacia, W. Macyk, How to correctly determine the band gap energy of modified semiconductor photocatalysts based on UV–Vis spectra. J. Phys. Chem. Lett. 9, 6814–6817 (2018). 30990726 10.1021/acs.jpclett.8b02892

[72] A. A. Govyadinov, I. Amenabar, F. Huth, P. Scott Carney, R. Hillenbrand, Quantitative measurement of local infrared absorption and dielectric function with tip-enhanced near-field microscopy. J. Phys. Chem. Lett. 4, 1526–1531 (2013). 26282309 10.1021/jz400453r

