# Supplementary material for: Correlated nanoimaging of structure and dynamics of cation-polaron coupling in hybrid perovskites
Source: Sci Adv. 2025 Feb 26;11(9):eads3706. doi: 10.1126/sciadv.ads3706 (PMC11864170; doi:10.1126/sciadv.ads3706)
Supplement: Supplementary file 1 — Supplementary Text Figs. S1 to S8 Tables S1 and S2 [file sciadv.ads3706_sm.pdf]

Science Advances
AAAS

Supplementary Materials for

Correlated nanoimaging of structure and dynamics of cation-polaron coupling in hybrid perovskites

Roland Wilcken *et al.*

Corresponding author: Markus B. Raschke, markus.raschke@colorado.edu

Sci. Adv. **11**, eads3706 (2025)
DOI: 10.1126/sciadv.ads3706

This PDF file includes:

Supplementary Text
Figs. S1 to S8
Tables S1 and S2

Perovskite film preparation

Fig. S1 shows additional information for the FADMACs film preparation. **Fig. S1B** shows the determination of the band gap by the Tauc method (*71*) for an assumed indirect band gap with the energy-dependent absorption coefficient α. For more information, please refer to the methods section in the main text.

Ground state vibrational nanospectroscopy

IR s-SNOM rater scans have been performed at 10 different locations of the sample around the formamidinium (FA) cation anharmonic stretch vibrational mode at $\bar{\nu}_0 \sim 1715\ \text{cm}^{-1}$ as described in the methods section. Examples are shown in **Fig. 2** in the main text. About 900 spectra in total have been measured and FA vibrational mode and the near field phase Φ_{NF} was fitted with an area-normalized Lorentz function to determine the amplitude A_L, center frequency $\bar{\nu}_0$ and linewidth Γ given by:

$$L(\bar{\nu}) = \frac{2A_L}{\pi} \cdot \frac{\Gamma}{4(\bar{\nu}-\bar{\nu}_0)^2+\Gamma^2} \tag{S1}$$

Note on ground and excited state peak fitting

The data are fit using a Lorentz profile for extraction of the peak position and linewidth. This process is reliable because the peak position as the centroid results from the statistics of the aggregate of data contributing to the full spectrum, and not just the few points defining the top of the peak. We derive an uncertainty for the peak position of ±0.5 cm^{-1} (compared to ±0.1 cm^{-1} for the ground state spectra).

What aids in the FA vibrational peak analysis is that the background, despite being large and structured, is not fluctuating, and several of the spectral features are a result of residual water in the system, despite extensive purging with dry nitrogen. This is assessed in repeated measurements at the same sample location showing the same spectral features in the background. We then typically average more than 20 interferograms at one location, reducing true noise by more than a factor of 4, i.e., close to the ideal value $\sqrt{N} = 4.5$. These variations of background between sample locations by and large do not affect the determination of peak position. Also, the same features just with larger FA to background contrast are observed in the ground state spectra.

Excited state vibrational nanospectroscopy

Vibrational Stark shift estimate and distance dependence

The estimate of the local electric field responsible for the observed vibrational Stark shift in the excited state shown in Fig. 3F is a model that demonstrates the necessity of a screening process that homogenizes the local field. To show this, we model a field E of a single unscreened charge at a distance r from the FA cation, i.e. $E = k \cdot e \cdot r^{-2}$ [Vcm^{-1}] (see **Fig. 3F**). Here k is the Coulomb constant, and e is the elementary charge. We calculate the corresponding Stark shift by dividing this electric field by an effective Stark tuning rate of $10.3\ \text{Vcm}^{-1}$ per wavenumber shift (*61*), providing the Stark shift as a function of distance between the unscreened electron and the FA cation (see **Fig. 3F**). This demonstrates that an unscreened electron would provide an electric field comparable to that required for the observed blue shifting. However, the fast decrease of the field strength (due to the r^{-2} dependance) and the equivalent reduction in the Stark shift would result in an inhomogeneous broadening of the observed FA spectrum, which is not observed. Therefore, we conclude that the additional field the FA cation is exposed to would be more

homogeneous, like in the case of a relatively uniform lattice distortion over larger distance (2 nm radius and more) a polaron induces (*14*).

Polaron recombination dynamics

We use three methods to fit the carrier decay dynamics at different locations on the sample. The stretched exponential fit and the rate model both use a time dependent recombination rate as the underlying model to account for the changing polaron density and polaron stabilization over time and are described in the main text. In principle, a multiexponential can be used to visualize the fast initial decay time τ_{fast} due to the high polaron density and the slower decay τ_{slow} at later delays when the density is reduced. The multiexponential model is given by:

$$A(t) = \sum_i A_{\text{i}} e^{-\frac{t}{\tau_i}} + y_0 \,, \tag{S2}$$

with amplitude A_{i}, time constant τ_{i}, and offset y_0. The multiexponential fit results in two time constants that vary spatially between τ_{fast} = 6 – 12 ps and τ_{slow} = 80 -150 ps. **Table S1** shows the fit parameter for the time traces shown in **fig. S4** (and in **Fig. 4D**). In general, all methods are consistent with showing faster time constants or higher rates, respectively.

Comparing the fast component of the multiexponential fit with the decay time constant of the excited state spectrum of 5.2 ps, they are within uncertainty. Beyond $T \gtrsim$ 20 ps, the signal-to-noise ratio becomes too low even with extensive averaging and the subsequent spectral decay is not resolved. Therefore, we conclude that the spectral decay follows the polaron recombination and the spectral signal should be proportional to the carrier density.

Correlations between ground state and excited state parameters

We attempt to compare ground state parameters and parameters for the excited state to see which one of them are correlated. The parameters for 15 sample positions where we measured the ground state spectrum, the excited state spectrum and the polaron decay curve for each are listed in **table S2**.

First, we repeat the correlation of ground state spectral parameters to each other (**see fig. S3**), namely the peak position $\bar{\nu}_0$, linewidth Γ and the amplitude A_{L} of a Lorentz fit to the anharmonic stretch vibration of the FA. Results are shown in **fig. S5**. Center frequency and linewidth are uncorrelated while there is weak correlation of both parameters with the amplitude. The amplitude increases if the local FA concentration is higher and therefore the structure of the perovskite is slightly different.

Next, we compare the parameters for the ground state FA spectrum with parameters from the excited state spectrum (namely the shift of the peak position $\Delta\bar{\nu}_0^*$, the linewidth in the excited state Γ^* and the linewidth change $\Delta\Gamma = \Gamma^* - \Gamma$) and with the characteristic numbers for the polaron decay (namely the average stretched exponential decay time $\langle\tau\rangle$ and fraction of the residual signal after 1 ns R_0). The respective plot including the Pearson correlation coefficients are shown in **fig. S6**. Most parameters seem to be weakly correlated or uncorrelated and we discuss a few interesting examples here.

The composition and therefore structure of the perovskite are reflected in the ground state frequency with a red shifted peak hinting to a "softer", easier polarizable lattice. We observe a moderate correlation between the ground state $\bar{\nu}_0$ and the excited state center frequency $\Delta\bar{\nu}_0^*$ of the FA cation vibrational mode (**fig. S6D, Fig. 5A**). Our interpretation is that the softer lattice leads to a larger deformation of the lattice from polarons and therefore a stronger local field giving a larger

spectral shift. In addition, we see a correlation between the peak position and the number of surviving polarons (**fig. S6C, Fig. 5B**). The softer lattice is likely to improve the polaron stabilization as well which makes them less likely to recombine. This directly links the structure of the perovskite with excited state spectral properties and carrier dynamics. The perovskite composition has an influence on the number of carriers available for photovoltaics. This corresponds to changes in the macroscopic composition, which has also a large influence on the material properties, but here we observe this effect on the nanoscale. Unfortunately, we cannot associate the center frequency with a certain composition, structure, or defect density.

An increase in the linewidth can have two causes. First, an inhomogeneous broadening due to local structural variations that exposes the FA cations to slightly different local fields which we refer to as disorder. Second, an increase in the cation-lattice interaction which reduces the dephasing time. We find a correlation between the ground state linewidth with the polaron decay time constant (**fig. S6E**). Our theory is that the linewidth increases due to larger local disorder and stronger cation-lattice coupling fosters polaron stabilization and decreases the recombination rate.

Most other parameters are rather weakly correlated although this is information as well. For example, the ground state amplitude (FA concentration) is not correlated with any other parameter. This means a change in FA concentration does not appreciably affect the structure, which is more likely influenced by the relative concentration of the other two cations in the FADMACs perovskite (*42*).

Lastly, we correlate all excited state parameters with each other (**fig. S7**). Here we see that excited state shift and polaron recombination are decoupled processes. Therefore, material composition and structure have a more profound influence than carrier induced transient effects.

Fluence dependence of the number of surviving carriers

From fluence dependent time traces (**fig. S8**) we determine the absolute number of polarons that survive the first ns (Abs. R_0) and the relative fraction of remaining carriers (Rel. R_0) compared to the initial carrier density, which we assume is proportional to heterodyne pump-probe amplitude R^*_{HPP}. With increasing pump-fluence the fraction of remaining carriers is reduced, since the initial carrier density is increased, and the bimolecular and Auger recombination become dominant. The absolute number of polarons instead is increased at higher fluences.

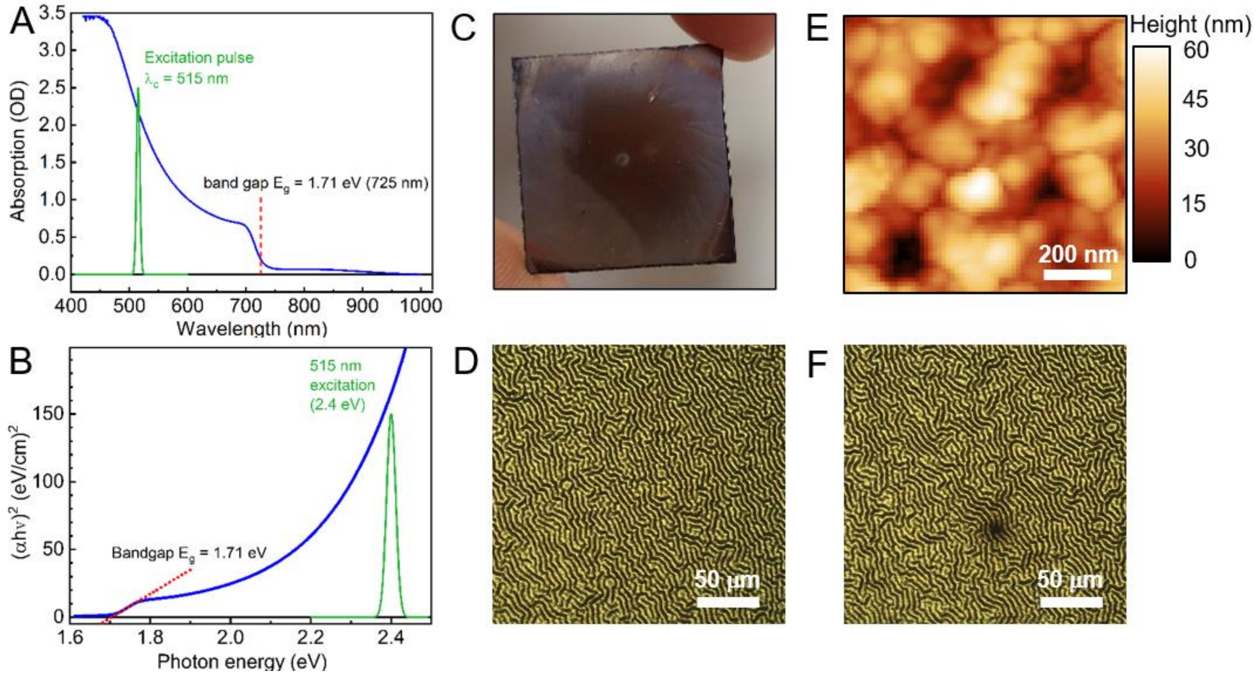

Fig. S1: Characterization of the FADMACs film. (A) Absorption spectrum of a 300 nm FADMACs film on a glass substrate. (B) Determination of the bandgap from absorption measurements by the Tauc method to 1.71 eV (compared to 1.73 eV (*41*). (C) Photo of the FADMACs film. (D) Optical microscope image of FADMACs film with visible Marangoni effect. (E) AFM topography in a 1x1 μm window with typical ~100 nm grains. (F) Optical microscope image of a burn spot on the surface after pump-probe measurements for several hours.

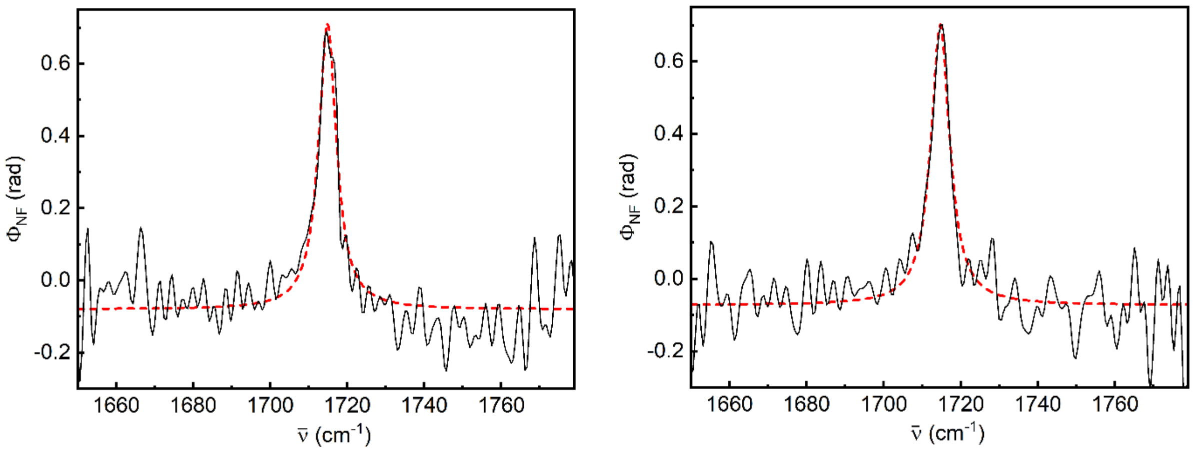

Fig. S2: Representative ground state spectra of the formamidinium (FA) cation vibration. Examples of nano-FTIR spectra of around the FA cation anharmonic stretch vibrational mode near field phase Φ_{NF} fitted with a Lorentz function.

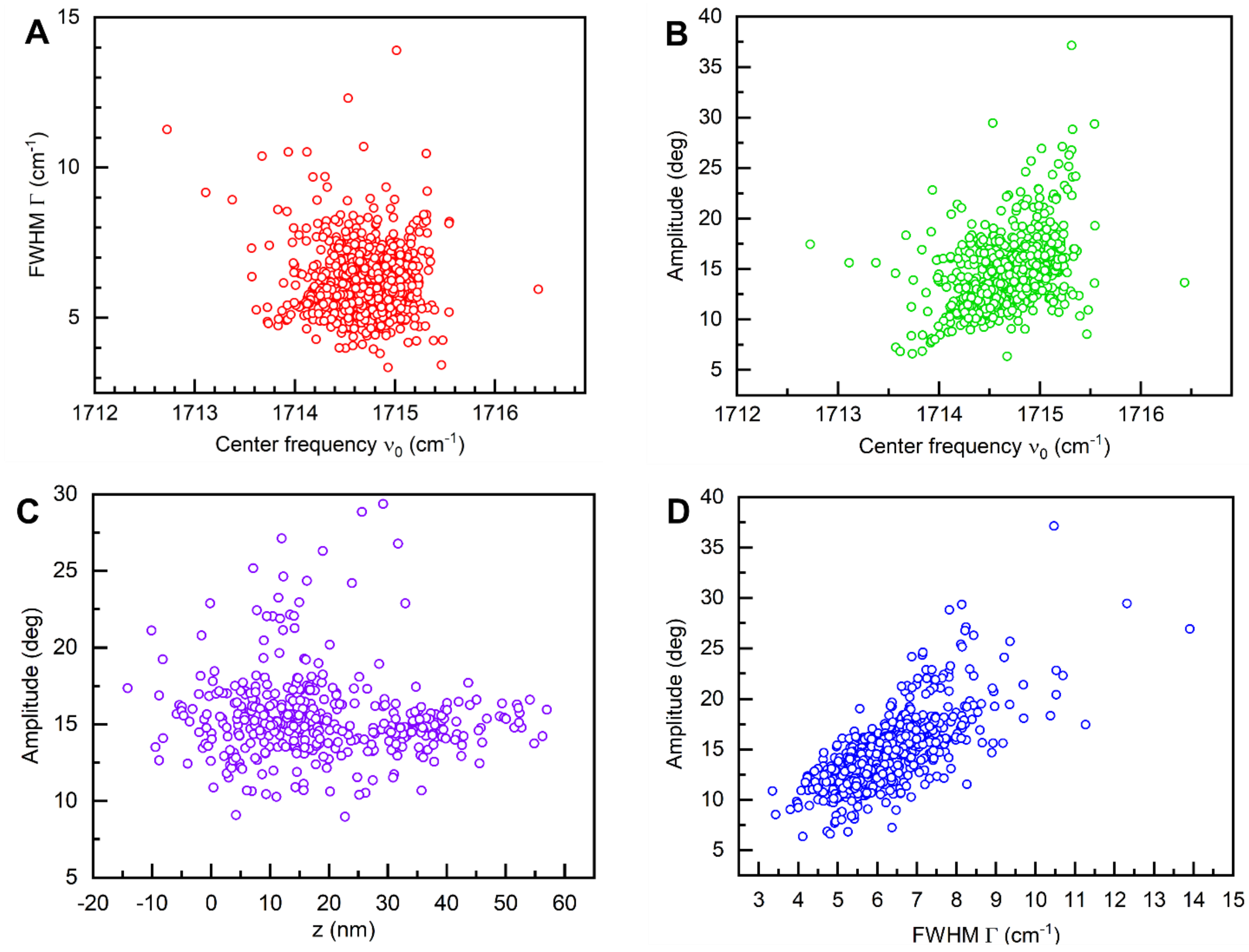

Fig. S3: Correlations between ground state characteristics. Results of 900 ground state nano-FTIR spectra of around the formamidinium cation anharmonic stretch vibrational mode fitted with a Lorentz function. (A) Linewidth Γ against center frequency $\bar{\nu}_0$. (B) Amplitude A_L against center frequency $\bar{\nu}_0$. (C) Amplitude A against topographic height z. (D) Amplitude against linewidth Γ.

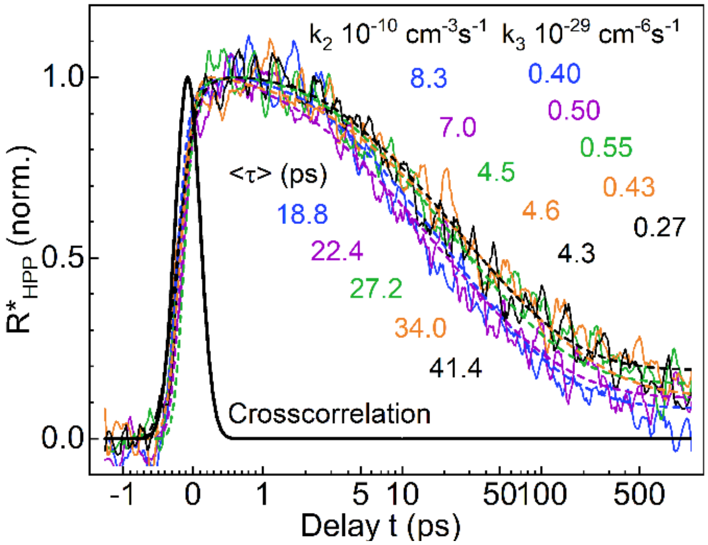

Fig. S4: Time traces at selected sample locations. 2-Phase heterodyne R^*_{HPP} time traces (solid lines) scanning the pump-probe delay T at 5 locations on the surface (see **Fig. 3B** for related spectra) fitted by a stretched exponential and the rate model.

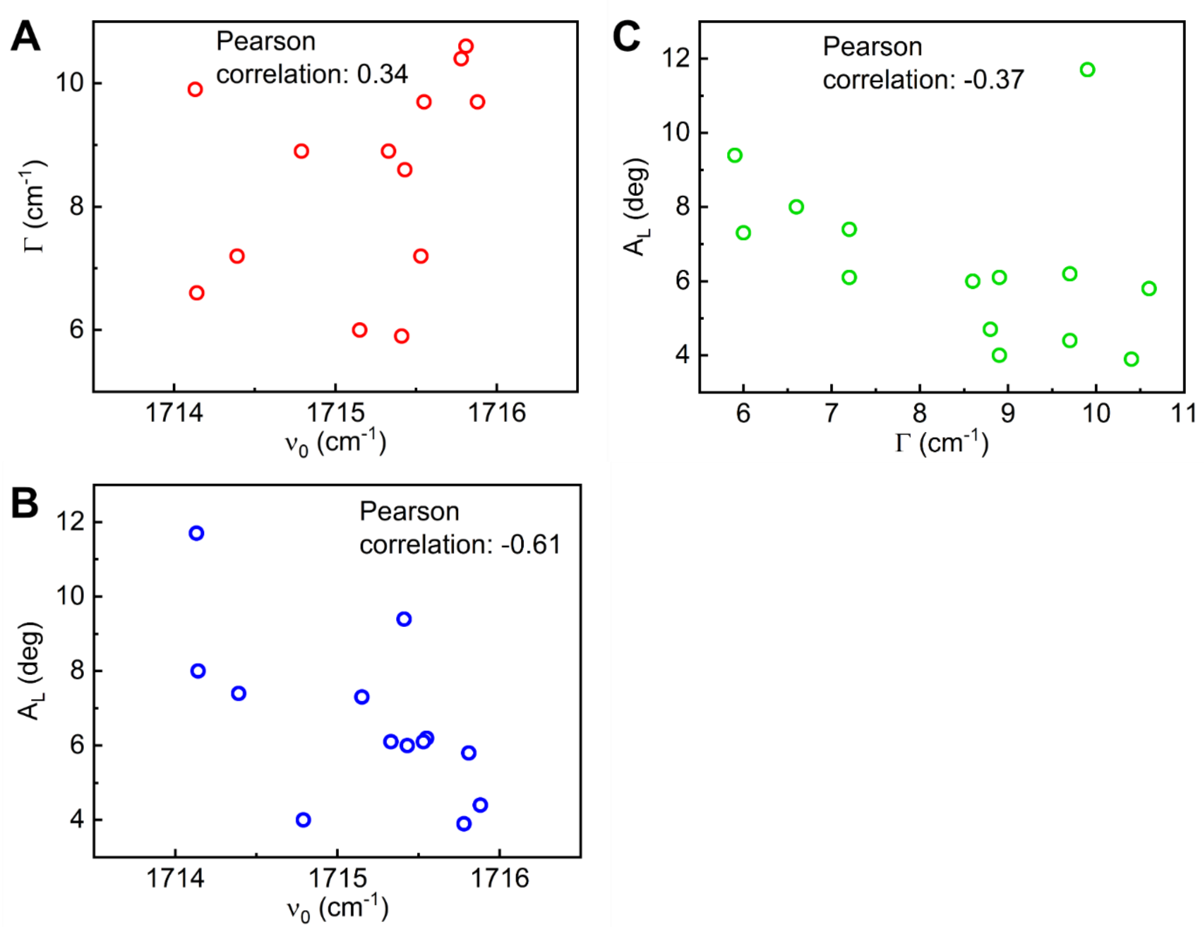

Fig. S5. **Correlations between ground state parameters in table S2.** Phase signal of the vibrational mode was fitted with a Lorentz model. (A) Ground state vibrational center frequency $\bar{v}_0$ against linewidth Γ. (B) Linewidth Γ against amplitude A_L. (C) Center frequency $\bar{v}_0$ against amplitude A_L. Pearson correlation coefficient shows the degree of correlation (0 means no correlation, 1 or -1 means strong correlation).

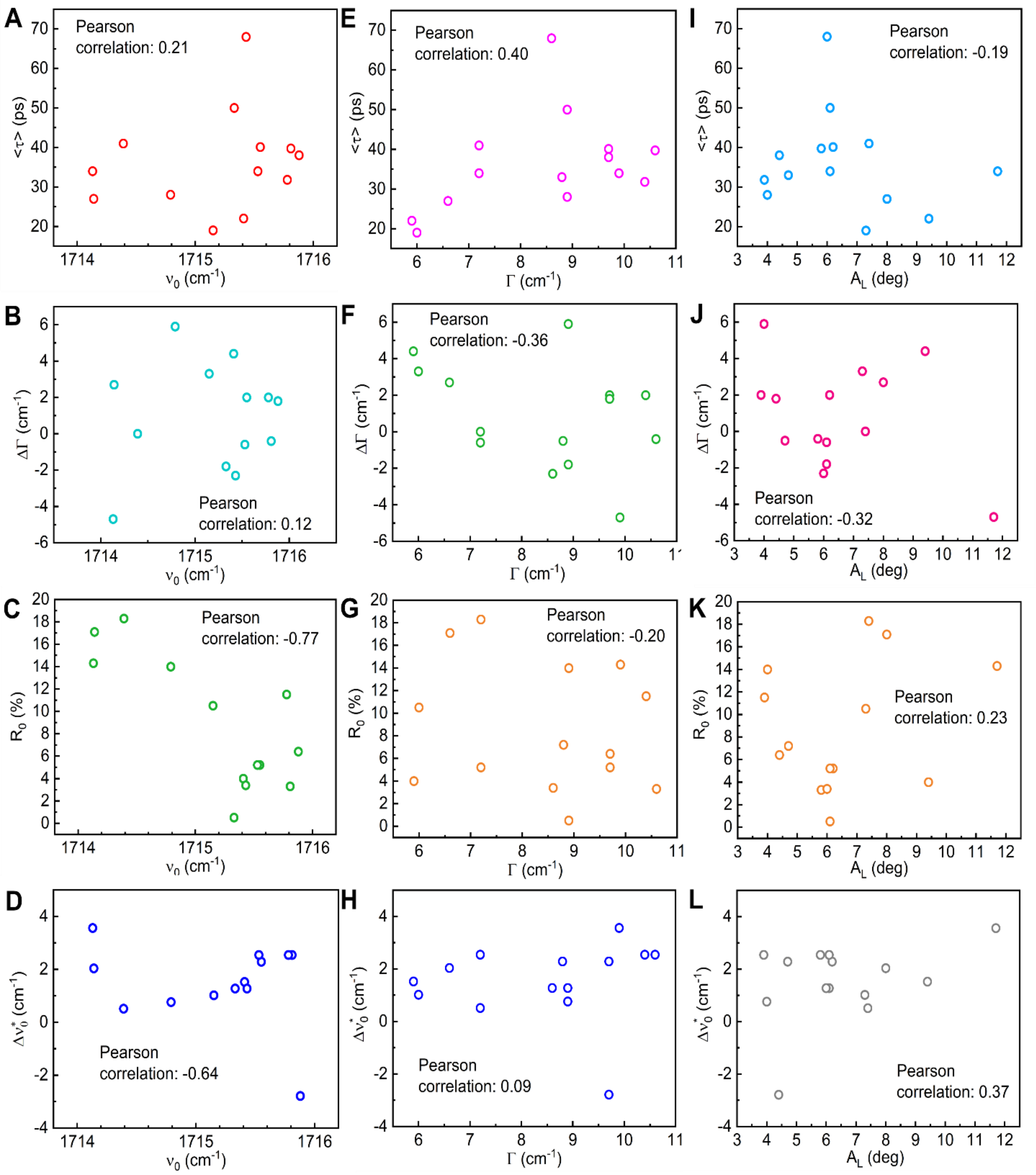

Fig. S6. Correlations between ground- and excited state parameters. Ground state center frequency $\bar{\nu}_0$ against average stretched exponential carrier decay time constant $\langle\tau\rangle$ (A), change in linewidth between ground and excites state $\Delta\Gamma$ (B), fraction of residual charge carriers after 1 ns R_0 (C), and spectral shift between ground and excited state $\Delta\bar{\nu}_0^*$ (D). Ground state vibrational mode linewidth Γ against time constant $\langle\tau\rangle$ (E), change in linewidth $\Delta\Gamma$ (F), residual carriers R_0 (G), and spectral shift $\Delta\bar{\nu}_0^*$ (H). Ground state vibrational mode peak amplitude A_L against time constant $\langle\tau\rangle$ (I), change in linewidth $\Delta\Gamma$ (J), residual carriers R_0 (K), and spectral shift $\Delta\bar{\nu}_0^*$ (L). Pearson correlation coefficient shows the degree of correlation (0 means no correlation, 1 or -1 means strong correlation).

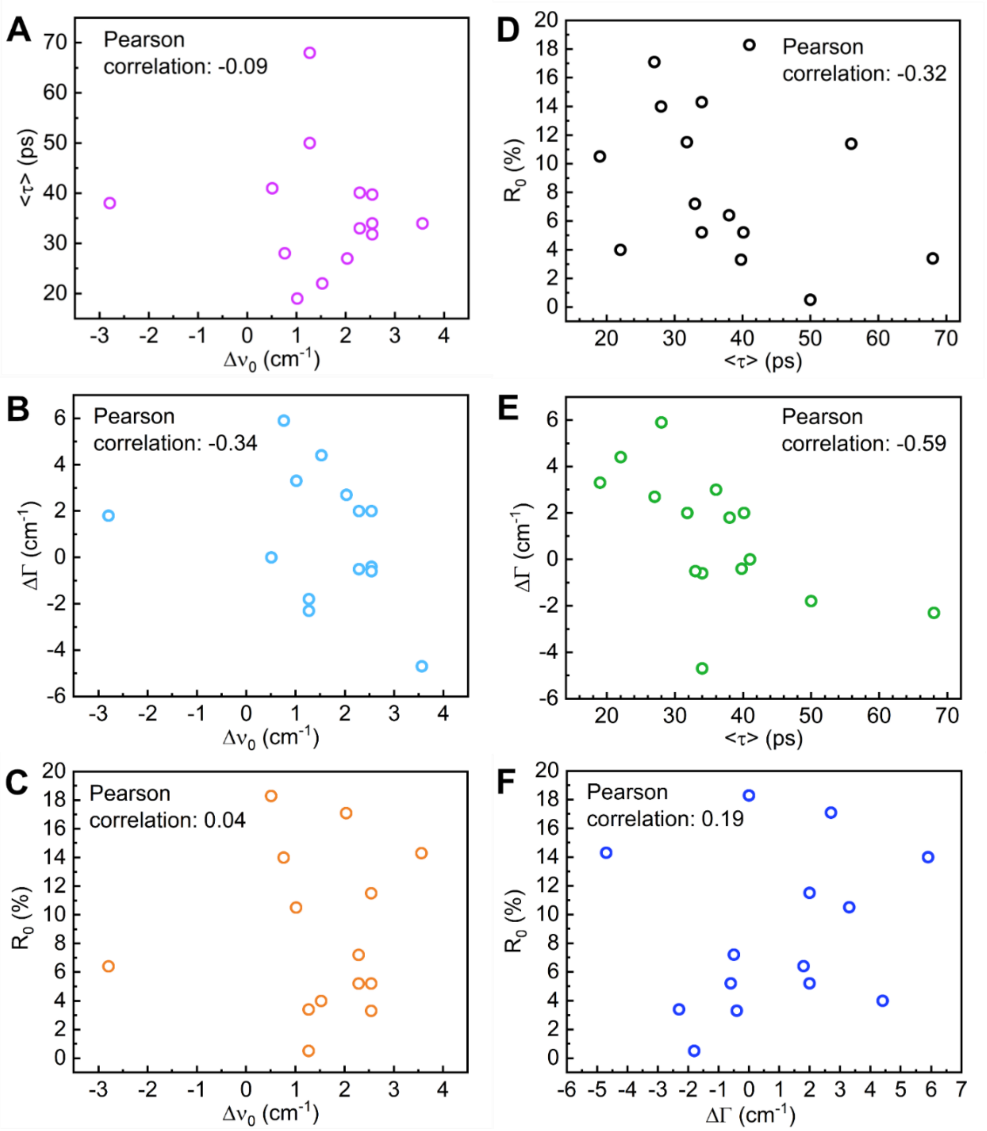

Fig. S7. Correlations between excited state parameters in table S2. Excited state spectral blue shift $\Delta\bar{\nu}_0^*$ against average stretched exponential carrier decay time constant $\langle\tau\rangle$ (A), change in the linewidth from ground to excited state $\Delta\Gamma$ (B), and fraction of residual carriers after 1 ns R_0 (C). Carrier decay time constant $\langle\tau\rangle$ against residual R_0 (D) and linewidth change $\Delta\Gamma$ (E). (F) Linewidth change $\Delta\Gamma$ against residual R_0. Pearson correlation coefficient shows the degree of correlation (0 means no correlation, 1 or -1 means strong correlation).

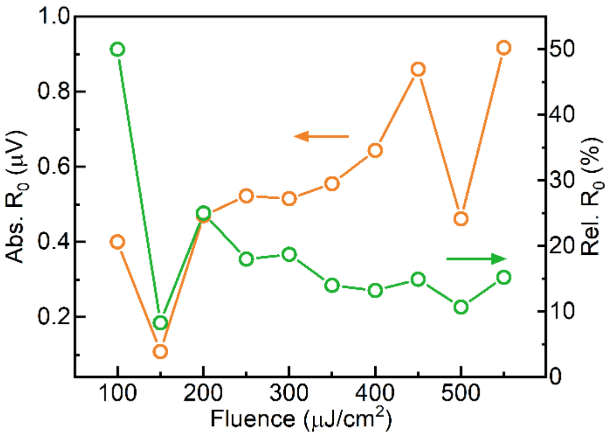

Fig. S8: Fluence dependence of the residual carrier population: Fluence dependence of the absolute (Abs. R_0) and relative fraction (Rel. R_0) of polarons after 1 ns.

**Pos.**	**A_{fast} (ps)**	**τ_{fast} (ps)**	**A_{slow} (ps)**	**τ_{slow} (ps)**	**$\langle\tau\rangle$ (ps)**	**k_2 (10^{-10} $cm^{-3}s^{-1}$)**	**k_3 (10^{-29} $cm^{-3}s^{-1}$)**	**R_0 (%)**
**1**	2.7	7.9	0.9	121	22	7.0	0.50	4
**2**	2.0	7.8	1.0	80	19	8.3	0.40	11
**3**	1.9	6.7	1.3	96	27	4.5	0.55	17
**4**	2.1	11.5	1.0	146	41	4.3	18	18
**5**	1.8	9.3	1.0	144	34	4.6	0.43	14

Table S1: Comparison of temporal fitting methods. Comparison of fit methods. Multiexponential fit with a two exponential time constant with the amplitudes A_{fast} and A_{slow} and the time constants τ_{fast} and τ_{slow}. Stretched exponential fit with the average time constant $\langle\tau\rangle$. Rate model fit with the bimolecular recombination rate k_2 and the many-body recombination rate k_3. R_0 is the fraction of remaining signal (~ proportional to the number of carriers) after 1 ns.

**Pos.**	$\bar{\nu}_0$ **(cm^{-1})**	Γ_0 **(cm^{-1})**	A_L **(deg)**	$\bar{\nu}_0^*$ **(cm^{-1})**	$\Delta\bar{\nu}_0^*$ **(cm^{-1})**	Γ^* **(cm^{-1})**	$\Delta\Gamma$ **(cm^{-1})**	A_L^* **(deg)**	$\langle\tau\rangle$ **(ps)**	R_0 **(%)**
**1**	1715.4	5.9	9.4	1717.9	1.5	10.3	4.4	23.7	22	4
**2**	1715.2	6.0	7.3	1716.2	1.0	9.3	3.3	20.7	19	11
**3**	1714.1	6.6	8	1716.1	2.0	9.3	2.7	15.2	27	17
**4**	1714.4	7.2	7.4	1714.9	0.5	7.2	0	14.6	41	18
**5**	1714.1	9.9	11.7	1717.7	3.6	5.2	-4.7	13.3	34	14
**6**	1715.8	10.6	5.8	1718.3	2.5	10.2	-0.4	6.5	40	3
**7**	1715.5	9.7	6.2	1717.8	2.3	11.7	2	15.6	40	5
**8**	1715.3	8.9	6.1	1716.6	1.3	7.1	-1.8	6.3	50	0.5
**9**	1715.5	7.2	6.1	1718.0	2.5	6.6	-0.6	18.2	34	5
**10**	1715.4	8.6	6	1716.7	1.3	6.3	-2.3	13.1	68	3
**(11)**	(1715.9)	(9.7)	(4.4)	(1713.1)	(-2.8)	(11.5)	(1.8)	(7.2)	(38)	(6)
**12**	1715.8	8.9	4	1716.6	0.8	14.8	5.9	7.5	28	14
**13**	1714.8	8.8	4.7	1717.1	2.3	8.3	-0.5	10.5	33	7
**14**	1715.8	10.4	3.9	1718.3	2.5	12.4	2	6.7	32	12
**(15)**	(1712.5)	(12.0)	(2.1)	(1719.4)	(6.9)	(16.5)	(4.5)	(10.1)	(36)	(6)

Table S2: **Ground and excited state fit parameters**. Ground and excited state parameters at 15 selected locations on the sample. Spectra and time traces for positions 1-5 are shown in **Fig. 3B** and **Fig. 4D**. Here $\bar{\nu}_0$ is the ground state center frequency, Γ_0 is the ground state linewidth, A_L is the peak amplitude, $\Delta\bar{\nu}_0^*$ is the excited state spectral blue shift of the FA cation vibrational mode, Γ^* is the excited state linewidth, $\Delta\Gamma$ is change in the linewidth from ground to excited state, A_L^* is the peak amplitude in the excited state, $\langle\tau\rangle$ is the average stretched exponential time constant for the polaron recombination and R_0 is the fraction of polarons surviving after 1 ns.
